# Optimizing chemistry at the surface of prodrug-loaded cellulose nanofibrils with MAS-DNP

**DOI:** 10.1038/s42004-023-00852-2

**Published:** 2023-03-28

**Authors:** Akshay Kumar, Bastien Watbled, Isabelle Baussanne, Sabine Hediger, Martine Demeunynck, Gaël De Paëpe

**Affiliations:** 1 Univ. Grenoble Alpes, CEA, CNRS, IRIG-MEM, Grenoble, France; 2 Univ. Grenoble Alpes, CNRS, DPM, Grenoble, France

**Keywords:** Solid-state NMR, Surface spectroscopy, Drug delivery, Solid-state NMR, Drug delivery

## Abstract

Studying the surface chemistry of functionalized cellulose nanofibrils at atomic scale is an ongoing challenge, mainly because FT-IR, NMR, XPS and RAMAN spectroscopy are limited in sensitivity or resolution. Herein, we show that dynamic nuclear polarization (DNP) enhanced ^13^C and ^15^N solid-state NMR is a uniquely suited technique to optimize the drug loading on nanocellulose using aqueous heterogenous chemistry. We compare the efficiency of two conventional coupling agents (DMTMM vs EDC/NHS) to bind a complex prodrug of ciprofloxacin designed for controlled drug release. Besides quantifying the drug grafting, we also evidence the challenge to control the concurrent prodrug adsorption and to optimize washing procedures. We notably highlight the presence of an unexpected prodrug cleavage mechanism triggered by carboxylates at the surface of the cellulose nanofibrils.

## Abstract (web summary)

Functionalized nanocellulose is a promising drug carrier, however, studying surface functionalization at the atomic scale remains challenging due to spectroscopic methodology limitations. Here, the authors use dynamic nuclear polarization enhanced ^13^C and ^15^N solid-state NMR to optimize the drug loading on nanocellulose using aqueous heterogeneous chemistry.

## Introduction

Nanocellulose has emerged as a class of bio-sourced and biodegradable material bearing many unique features. Beyond its non-toxicity and its high mechanical stability, its large surface area (50–150 m^2^/g) makes it an interesting nanoplatform for chemistry with a high degree of flexibility. As such, cellulose nano crystals (CNC) and cellulose nanofibrils (CNF) have gained increasing attention resulting in >5000 peer-reviewed articles in the last 5 years.

Cellulose nanomaterials (CNMs) are currently being developed as composite reinforcement fibers and fillers for concrete, automotive, and packaging industry. Beyond these high-volume applications, CNMs are also developed as a sustainable platform for numerous emerging energy and health-related applications^1–5^. In the field of pharmaceuticals, powdered CNCs/CNFs are already widely used as pharmaceutical excipient, i.e., as tablet binder and diluent. In addition, many formulation strategies are also being developed (e.g., spray-drying) with the aim to improve the sustained release of the drug nanocrystals entrapped in the CNF network. CNFs have also been recently proposed as promising drug carrier with potential for controlled release activity^6–9^. More specifically, antibacterial agents or active pharmaceutical ingredients have been loaded into CNF gels to develop innovative antibacterial materials and smart drug carriers^8,10–13^. The latter approach is particularly interesting when the active molecule is poorly soluble (which limits its absorption along the gastrointestinal tract) and/or there is a need to limit off-target activity of the drug because of severe side-effects (e.g., oncologic drug development)^14–17^. To this end, one can envision loading the prodrug either through adsorption or covalent grafting on the CNF surface, and then releasing the drug by enzymatic activation^14,18,19^.

So far, qualitative characterization of modified nanocellulose materials is being performed using various spectroscopic techniques including FTIR, XPS, Raman spectroscopy, and solution and solid-state NMR (ssNMR)^20^. However, as highlighted by Foster et al.^20^, these tools often lack sensitivity and/or resolution to unambiguously characterize CNF surface modification, especially for <5 wt% loading.

This limitation was also recently highlighted in our work where we showed that Dynamic Nuclear Polarization (DNP) enhanced solid-state NMR, a hyperpolarization technique, was uniquely suited to report atomic-scale information related to the surface chemistry of functionalized TEMPO-oxidized CNFs (referred to as CNF-t)^21^. TEMPO-oxidation of CNF is a common procedure pioneered by Isogai and co-workers^22,23^ to introduce carboxylic groups on the surface of CNF for further functionalization of the material. Beyond providing a precise estimation of the 1 wt% loading of an antibiotic drug (metronidazole) while differentiating between adsorption and covalent grafting, we showed that Magic Angle Spinning Dynamic Nuclear Polarization (MAS DNP) could also be used to understand the chemistry of heterogeneous reaction. This work has highlighted the need for quantitative atomic-scale information on such systems and the limitations of techniques such as conductometric titration and elemental analysis in this regard. It also paves the way for the rational design of drug carrier assisted by DNP-enhanced NMR. The quantitative aspect of the analysis was notably achieved through the use of multiCP^24^ based MAS DNP experiments. Although the use of multiCP was already reported in the context of DNP experiments^25–27^, our work proved its relevance for the quantitative study of surface-modified cellulose materials and thus confirms its broad applicability, as also seen in several recent studies^28–33^.

Building on this result, we now discuss a general strategy, based on the use of DNP-enhanced solid-state NMR, to optimize and control prodrug loading onto CNF-t. Heterogeneous chemistry on cellulose nanomaterial typically relies on many experimental parameters which are often critical to adjust due to the lack of atomic-scale information. Besides, synthetic procedures, monitoring the reaction progress, and washing steps cannot simply be extrapolated from analogous homogeneous reactions, and likely depend on the prodrug chemical characteristics and also on the CNF-t surface features. The presented methodology is applied to the ciprofloxacin prodrug for which the linker has been designed for controlled drug release. Indeed, the linker possesses an ester bond that can be cleaved in presence of esterase enzyme to release the desired ciprofloxacin antibiotic drug. We first discuss the choice of coupling agents for the amidation reaction and specifically compare the use of ethyl-3-(3-dimethylaminopropyl) carbodiimide (EDC) and *N*-hydroxysuccinimide (NHS) with 4-(4,6-dimethoxy-1,3,5-triazin-2-yl)-4-methylmorpholinium chloride (DMTMM) as an alternative coupling reagent. Then we show that beyond detecting prodrug loading, data collected with DNP can be used to differentiate and quantify adsorption versus covalent binding. This approach can therefore be used to determine the most effective coupling agents for functionalizing CNFs, while assessing the removal of excess reagents and other residual coupling agent side-products from the CNF surface. Finally, the approach highlights the importance and critical effect of pH during the washing steps. It can indeed modulate the efficiency of excess/adsorbed prodrug removal, as well as the unforeseen possibility of prodrug hydrolysis via a surface-catalyzed mechanism.

## Results

### Heterogeneous amidation onto CNF-t: EDC/NHS versus DMTMM as coupling agent

In our previous work, in which tempo-oxidized CNF (CNF-t) was functionalized with an antibiotic (modified metronidazole prodrug)^21^, we evidenced the presence of adsorbed coupling agents, EDC and NHS, as well as the grafting of possible by-products on the surface, e.g. the formation of EDC-*N*-acylurea^34^. These residual by-products could not be removed even after several cycles of washing/centrifugation followed by dialysis. Such unwanted side-reaction limits the maximum possible grafting of the prodrug and leads to the presence of undesirable products in the drug carrier. As a result, the identification and removal of excess reagents and coupling agents, whether bound or unbound, is a critical issue to address.

We use here DNP-enhanced ssNMR to investigate DMTMM as an alternative coupling agent to EDC/NHS with the aim of limiting the presence of unwanted covalently bound by-products of coupling agents onto the CNF surface. It is worth noting that in a recent article by Hoenders et al.^35^, the presence of by-products of EDC (as EDC-*N*-acylurea) was questioned after chemical modification of nanocellulose. This led the authors to use DMTMM as coupling agent. Interestingly, a recent study by D’Este et al.^36^ compared the efficacy of DMTMM vs EDC/NHS in the case of hyaluronan ligation via amidation in water. This study, conducted under homogenous conditions, concluded that DMTMM was a better choice. It is therefore very interesting to investigate whether this still applies under heterogeneous conditions *(vide infra*).

**Fig. 1:**
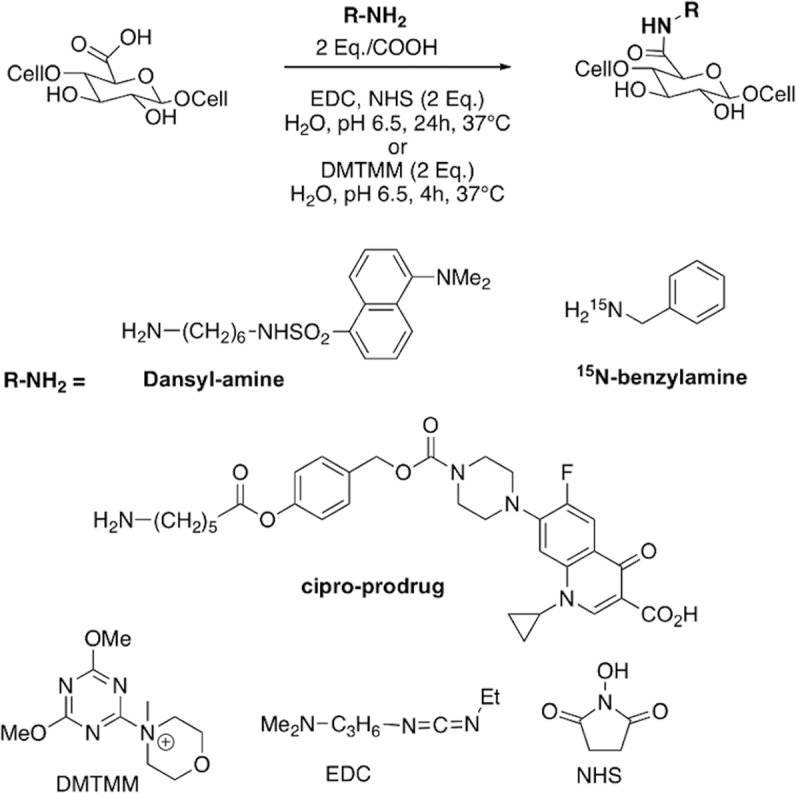
Amidation reaction and coupling agents. Covalent binding of various amino-substituted compounds (Dansyl-amine, ^15^N-benzylamine, cipro-prodrug) on CNF-t using either EDC/NHS or DMTMM as coupling agents. Chemical structures of the coupling agents are given at the bottom of the figure.

To evaluate the relevance of using DMTMM instead of EDC/NHS for CNF-t functionalization, we performed a comparative study using *N-*(6-aminohexyl)-5-dimethylamino-naphthalene-1-sulfonamide as fluorescent simple model (referred to as dansyl-amine in this work). The fluorescence of the chromophore was used to monitor the progress of the amidation reaction (see Fig. 1) and to optimize the purification steps. The reaction product is referred to as CNF-dansyl/EDC-NHS or CNF-dansyl/DMTMM when EDC/NHS or DMTMM was used as coupling agents, respectively. The reactions were performed in deionized water at a pH of 6.5, kept at 37 °C, using a two-fold excess of coupling agents relative to carboxylates.

After completion of the coupling reaction, several washing/centrifugation cycles were performed until no residual fluorescence could be observed in the supernatant. The fluorescence was directly checked in the supernatant using a portable UV/vis lamp irradiating at 254 and 365 nm. Washings were first carried out under acidic conditions (aqueous HCl, pH 3) in order to protonate the excess of amines (from the unreacted reagent and EDC or DMTMM coupling agents), as well as the residual carboxylates present on the surface of the CNFs. This step potentially allows to dissociate the ammonium salts interacting with the surface carboxylate functions. A total of five acid washings were needed to remove any trace of unreacted dansyl reagent. Subsequently, several washings with distilled water were carried out until the suspensions were back to near neutral pH. A final centrifugation was performed with each sample and the pellets were lyophilized to obtain white fluorescent solids.

### Atomic-scale information on the surface chemistry using DNP-enhanced solid-state NMR

Following our recent work, we used Magic Angle Spinning Dynamic Nuclear Polarization (MAS-DNP) to record sensitivity-enhanced solid-state NMR spectra of the functionalized CNF samples. MAS-DNP has recently become a key NMR approach to study with unprecedented sensitivity and thus atomistic details various types of systems from functional material to complex biomolecular systems^37–41^, including cellulosic materials^21,42–48^. This approach complements standard ssNMR experiments which are routinely used to characterize nanocellulose but have also been shown to be limited in terms of sensitivity, especially to detect low weight percent chemical modifications on the surface of CNMs^20^.

DNP relies on the combined use of state-of-the-art equipment^49^ (high-frequency source, low-temperature MAS probe, etc.) and the development of efficient (paramagnetic) polarizing agents^50–58^, which serve as source of hyperpolarization. Under suitable conditions, the large electron spin polarization is transferred to the surrounding nuclei, resulting in large gains in sensitivity^59–61^. This can typically enhance NMR sensitivity by several orders of magnitude, enabling new experiments which were not feasible so far. In particular, the development of DNP has made it possible the study of various materials at natural isotopic abundance^30,45,62,63^, and hence is being seen as a new era in material characterization^64^.

**Fig. 2:**
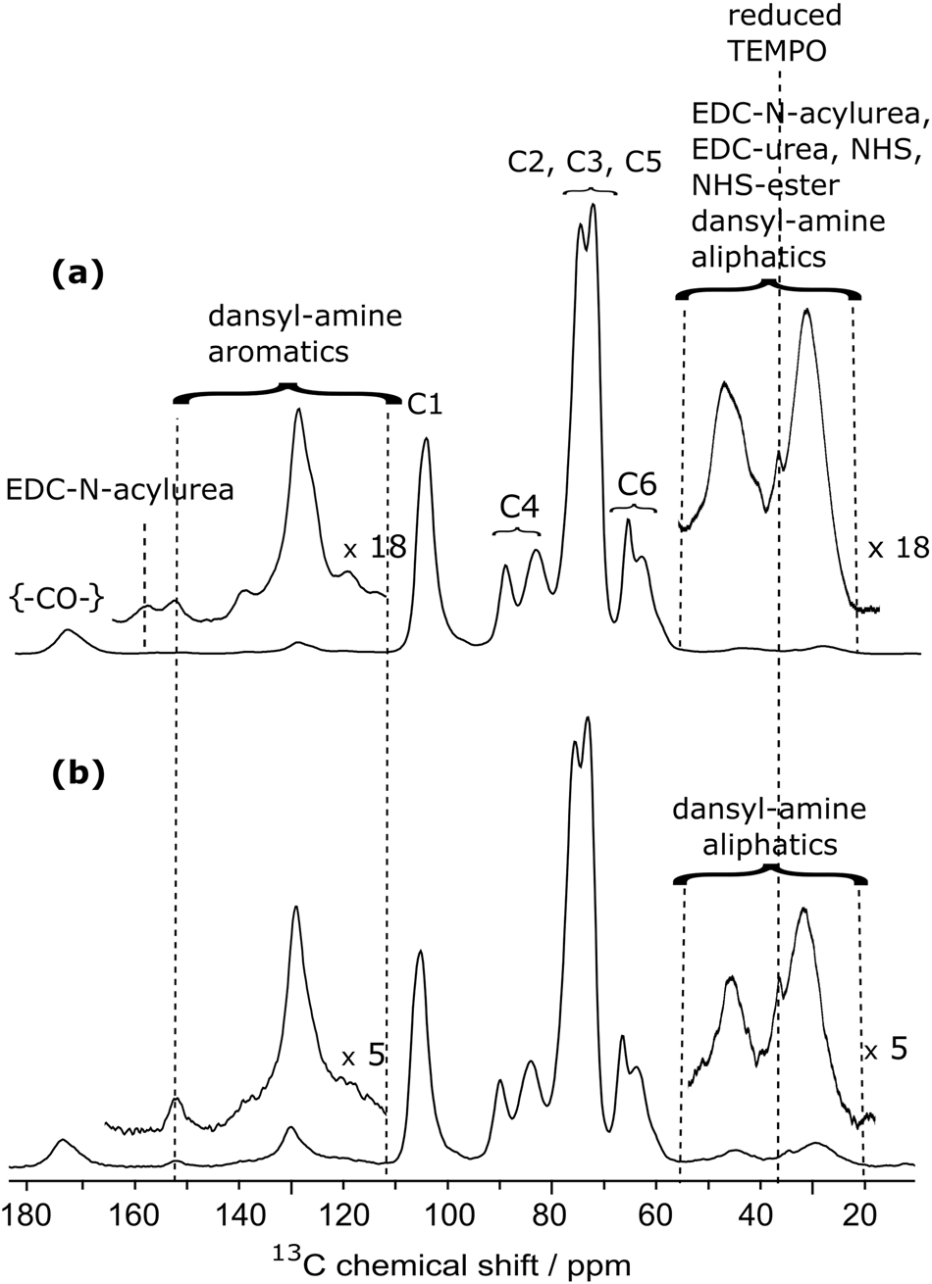
DNP-enhanced 13C NMR of surface-functionalized CNF. DNP-enhanced {^1^H}-^1**3**^C CPMAS NMR spectra of surface-functionalized CNF with 6-aminohexyl dansyl-amide using **a** EDC and NHS and **b** DMTMM as coupling agent. The ^13^C resonance assignment for (**a**) and (**b**) is given in the figure. The insets in (**a**) and (**b**) show magnified views of the 20–55 ppm and 115–165 ppm regions. Both spectra were normalized with respect to the cellulose signal intensities. Magnification factors for the insets, ×18 for (**a**) and ×5 for (**b**), were chosen in order to match the signal intensity for the aromatic dansyl-amine signals.

DNP-enhanced ^13^C cross-polarization experiments under MAS (CPMAS) were performed on CNF-dansyl/EDC-NHS and CNF-dansyl/DMTMM (Fig. 2). In both cases, a gain of ~25 in sensitivity was achieved compared to conventional NMR experiments. In addition to the broad resonances from cellulose (C1-C6) between 60 and 110 ppm and the surface carboxylic acids near 175 ppm, one can observe additional signals in the aromatic (115 to 165 ppm) and aliphatic (20 to 55 ppm) regions in both spectra. It should be noted that no aldehyde signal (expected near 185–200 ppm) is observed in either spectrum.

**Fig. 3:**
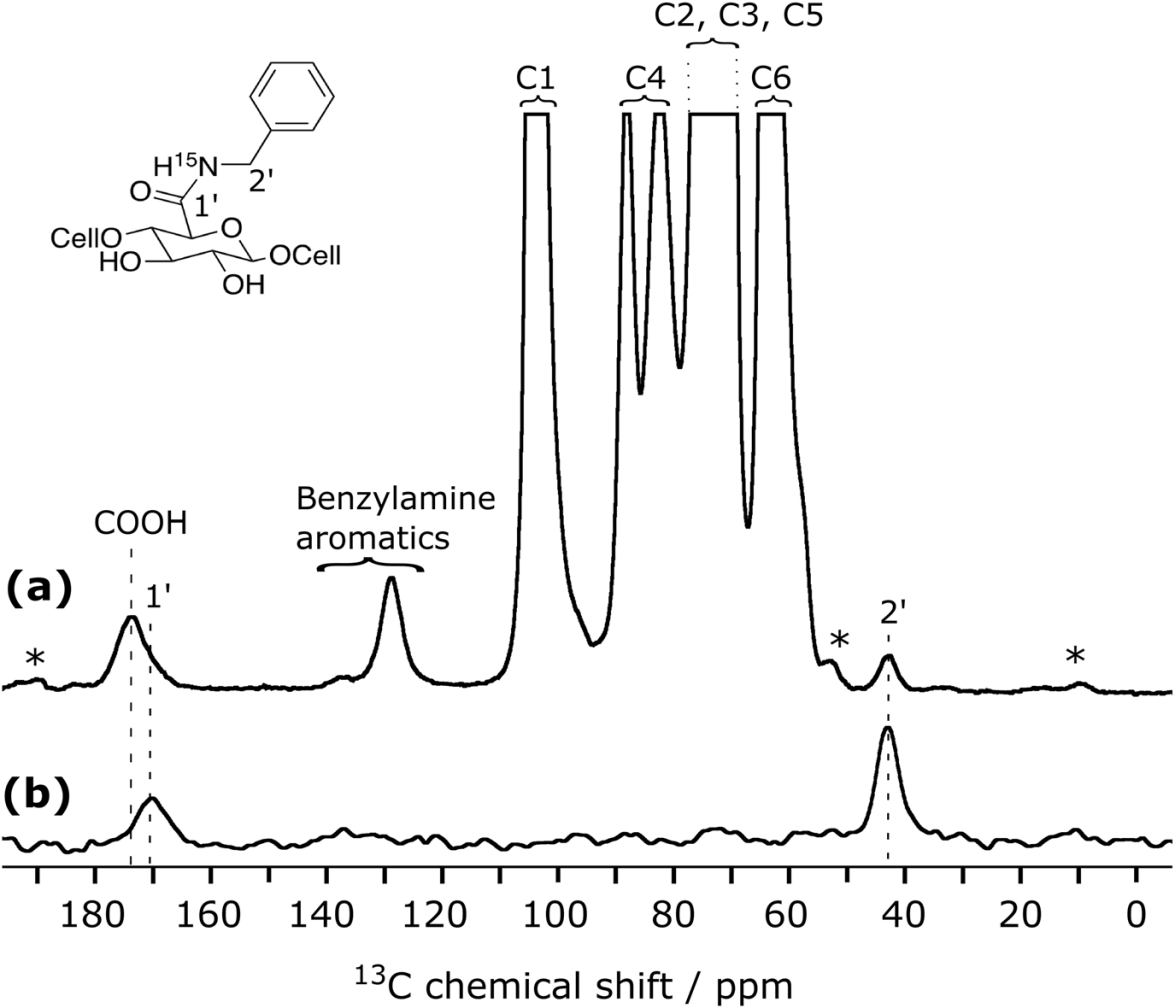
DNP-enhanced {15N}-13C NMR of surface-functionalized CNF. DNP-enhanced **a** {^1^H}-^13^C CPMAS spectrum and **b** {^15^N}-^13^C TEDOR spectrum of ^15^N-enriched benzylamine functionalized onto CNF. The resonance at 170 ppm in (**b**) confirms the amide bond formation. Resonance at 175 ppm in (**a**) is assigned to the unreacted carboxylic acid groups. Asterisks indicate spinning sidebands.

Resonance assignment of non-cellulosic resonances is not straightforward since multiple species contribute to the signal in these regions. From the spectral deconvolution of the resonances present between 115 and 155 ppm (see Fig. S2) and their relative integration, it results that 10 aromatic carbons are present, which we assigned to the aromatic carbons of the dansyl moiety. The broad resonance between 156 and 162 ppm, only present in the CNF-dansyl/EDC-NHS spectrum (Fig. 2a), can be attributed to EDC by-products, EDC-*N*-acylurea and EDC-urea^21^. Grafted/adsorbed amines are contributing to the signal in the aliphatic region between 20 and 55 ppm (i.e., two methyls of dansyl-amine and the -CH_2_- of the hexyl). The relative signal intensity in this region is higher for CNF-dansyl/EDC-NHS than for CNF-dansyl/DMTMM, as a result of additional contributions from EDC by-products (EDC-urea and EDC-*N*-acylurea), as well as NHS/NHS ester. Furthermore, one can also observe signal from the reduced TEMPO moiety in both spectra at 38 ppm. Note that this resonance is already present in the starting material CNF-t. Resonances assigned so far to dansyl-amine do not allow distinguishing between species adsorbed and/or chemically grafted on the surface. The fine analysis of the ^13^C signal between 165 ppm and 185 ppm should afford a unique opportunity to assess the situation. It appears as a broad multi-component peak, with a dominant contribution centered at ~175 ppm and shoulders resulting from components at lower frequencies (~172 and 170 ppm). The carboxylic acid groups can be assigned to the resonance at 175 ppm while the amide function is expected at ~170 ppm. The latter was confirmed by grafting ^15^N-labeled benzylamine using DMTMM. This sample (referred to as CNF-^15^*N*-benzylamine) was used as model compound to record ^15^N-^13^C dipolar correlation experiments. The ^15^N-filtered ^13^C TEDOR^65^ spectrum displayed in Fig. 3b contains only signals from ^13^C nuclei that are close in space to a ^15^N spin, which unambiguously confirms the assignment of the amide bond at 170 ppm. TEDOR stands for transferred echo double resonance and refers to a pulse sequence that can probe ^15^N-^13^C distance information.

### Quantifying CNF surface species

**Fig. 4:**
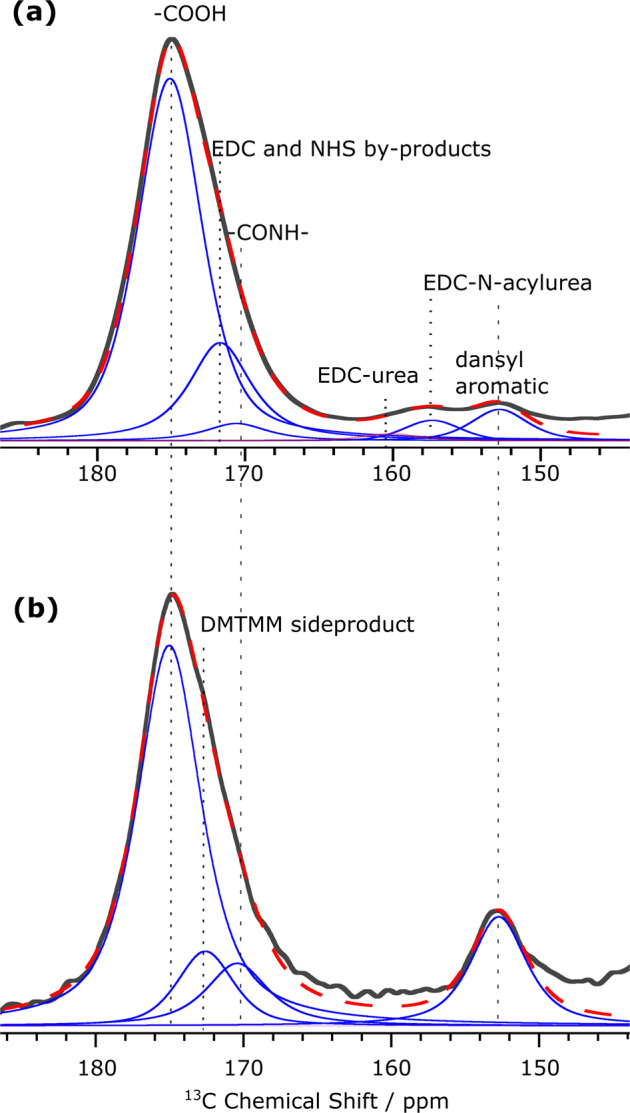
DNP-enhanced 13C NMR of surface-functionalized CNF. Signal deconvolution of the region between 145 and 185 ppm in ^1**3**^C DNP-enhanced CPMAS spectra (in black) of **a** CNF-dansyl/EDC-NHS and **b** CNF-dansyl/DMTMM. The deconvolution components are shown in blue and their sum in red (dashed line). Assignment is indicated in the figure. DMTMM side product refers here to DMT-OH.

To quantify the CNF surface species, we performed a deconvolution of the 145 to 185 ppm region of the two CNF-dansyl spectra (CNF- dansyl/EDC-NHS and CNF-dansyl/DMTMM in Fig. 4a, b, respectively). This deconvolution, within part components of small intensity, was only possible thanks to the high sensitivity provided by DNP. The signal component at 153 ppm, present in both spectra, corresponds to one of the dansyl aromatic resonances. The amide bond and the unreacted carboxylic groups are observed at 170 ppm and 175 ppm, respectively. Besides these resonances, a third carbonyl component is observed at 172 and 173 ppm in Fig. 4a, b, respectively. Following our recent work^21^, the contribution at 172 ppm, present in the EDC/NHS case (Fig. 4a), is assigned to coupling agent derivatives such as EDC-*N*-acylurea, NHS-ester, and possibly NHS. Other resonances from EDC derivatives are also observed at 157 and 161 ppm in Fig. 4a. The resonance at 173 ppm in Fig. 4b can be assigned to the triazine aromatic carbons present in the 2,4-dimethoxy-6-hydroxy-1,3,5-triazine (DMT-OH), which is a side-product of DMTMM (Fig. S3).

The DNP enhancement being uniform across all peaks in each spectra, both in terms of *ε*_on/off_ and polarization build-up, we can estimate the amount of the different species in the sample with respect to the number of CNF glucose units based on the proposed resonance assignment and the corresponding deconvoluted peak integrals. It is to note that CPMAS experiments are intrinsically not quantitative as CP spin-dynamics can vary for different types of carbon species, e.g., between protonated methyl and quaternary carbon sites. However, since both samples contain similar chemical species and were run with exactly same parameters, a semi-quantitative comparison can reasonably be performed and is presented in Table 1. It clearly suggests that DMTMM is better performing as coupling agent than EDC/NHS. Indeed, dansyl grafting is about four times more efficient with DMTMM. In addition, the amount of residual coupling agents and derivatives remaining on the CNF surface is smaller in the DMTMM case. Interestingly, the presence of grafted coupling agents on the CNF surface when using EDC/NHS (as opposed to DMTMM) could partly explain why the dansyl grafting efficiency is smaller. Based on these results, we chose to use DMTMM as coupling agent for all further reactions.

**Table 1:** Estimation of the relative amount of the different species present at the surface of CNF-t, CNF-dansyl/EDC-NHS, and CNF-dansyl/DMTMM with respect to the number of cellulose units.

Signal integral^a^	CNF-t	CNF-dansyl/EDC-NHS	CNF-dansyl/DMTMM
C4 (total)	100	100	100
CO (total)	25.0 ± 1.2	26.5 ± 1.3	30 ± 3
Dansyl-amine (total)	na	1.3 ± 0.1	5.5 ± 0.6
Bonded dansyl-amine	na	0.9 ± 0.1	5.5 ± 0.6
Grafted coupling agent derivatives	na	3.9 ± 0.2^b^	na
Adsorbed coupling agent side product^c^	na	0.3 ± 0.1	2.0 ± 0.2
Unreacted CO	25.0 ± 1.2	22.0 ± 1.1	17.8 ± 1.8

Error bars were estimated at 5% for CNF-t and CNF-dansyl/EDC-NHS, and 10% for CNF-dansyl/DMTMM. Larger error for CNF-dansyl/DMTMM is explained by the lower signal-to-noise ratio of the spectrum.

^a^All peak integrals reported in the table were normalized to the corresponding C4 peak integral between 82 and 94 ppm (containing both the narrow and the broad resonances of C4) set to 100.

^b^Calculated by integrating the deconvolution component at 172 ppm (Fig. 4a), which is mainly coming from EDC-N-acylurea. Contribution from NHS (bonded or adsorbed) cannot be excluded.

^c^Calculated by integrating the deconvolution component at 161 ppm (corresponding to EDC-urea) for CNF-dansyl/EDC-NHS (Fig. 4a) and at 173 ppm (corresponding to DMTMM side product) for CNF-dansyl/DMTMM (Fig. 4b).

### Grafting a prodrug onto CNF-t through amidation: the case of ciprofloxacin

To further illustrate the potential of DNP-enhanced NMR in the detailed analysis of functionalized CNMs, we extended the approach to the case of a large hydrophobic molecule, namely the ciprofloxacin prodrug (cipro-prodrug). The corresponding structure is depicted in Fig. 1 and its synthesis described in the SI (see Supplementary Methods and Scheme 1).

Ciprofloxacin is a widely used antibiotic. To limit its side-effects, various prodrugs have been prepared as reported in literature^66,67^. We chose to link an amine-terminated cleavable linker to the ciprofloxacin piperazine nitrogen by a carbamate bond. The terminal primary amine was then used to bind the prodrug on the CNF-t surface by reaction with the carboxylic acid groups (Fig. 1). The linker contains a phenol derived activated ester that, upon treatment with esterase enzyme or under acid/base catalysis, will release the drug.

### Investigating the coupling reaction of ciprofloxacin prodrug and the washing procedure

Coupling reactions were performed under the conditions described above for dansyl-amine. The modified CNF was then subjected to acidic washing steps (pH 3, diluted aqueous HCl) in order to favor the release of free or (weakly) adsorbed amine containing products. Unlike what was observed previously with dansyl-amine, a continuous release of the fluorescent ciprofloxacin chromophore was observed, even after 5 acidic washing/centrifugation cycles. Four additional washings with distilled water (pH 6) were then performed, but fluorescence was still observed in each supernatant. The resulting CNF-prodrug is referred here to as CNF-prodrug-pH3/6. Considering a possible release of the ciprofloxacin drug by acid-catalyzed hydrolysis of the ester bond, we prepared a second batch of CNF-prodrug, named here CNF-prodrug-pH6, under the same reaction conditions, but using only distilled water for the washing/centrifugation cycles. Surprisingly, in this case as well, fluorescence could still be observed in the supernatant after 10 washing/centrifugation cycles.

To understand the release phenomenon occurring during the washing steps, we recorded the fluorescence spectrum of the collected aqueous phases of each sample (CNF-prodrug-pH3/6 and CNF-prodrug-pH6) and compared them with the spectrum of the unbound starting cipro-prodrug dissolved in water (see Fig. S1). Clearly, the fluorescence spectra of the two CNF samples are different and neither of them matches the spectrum of the starting cipro-prodrug. The later shows one emission band around 400 nm, whereas ciprofloxacin is generally found around 446 nm, depending on the medium and pH^68^. The CNF-prodrug-pH6 spectrum displays one emission band at 444 nm, which is thus not consistent with the presence in the supernatant of the prodrug itself but rather of ciprofloxacin. CNF-prodrug-pH3/6 supernatant gives rise to two emission bands at around 400 and 463 nm, which is consistent with the release of both the cipro-prodrug and ciprofloxacin. This is probably related to the two-step washing procedure, in which the acidic washings were followed by neutral washings. These puzzling results were further investigated at the atomic scale using DNP-enhanced NMR to support the above hypothesis.

### Adsorption versus covalent grafting of a prodrug onto CNF probed by DNP-enhanced ssNMR

**Fig. 5:**
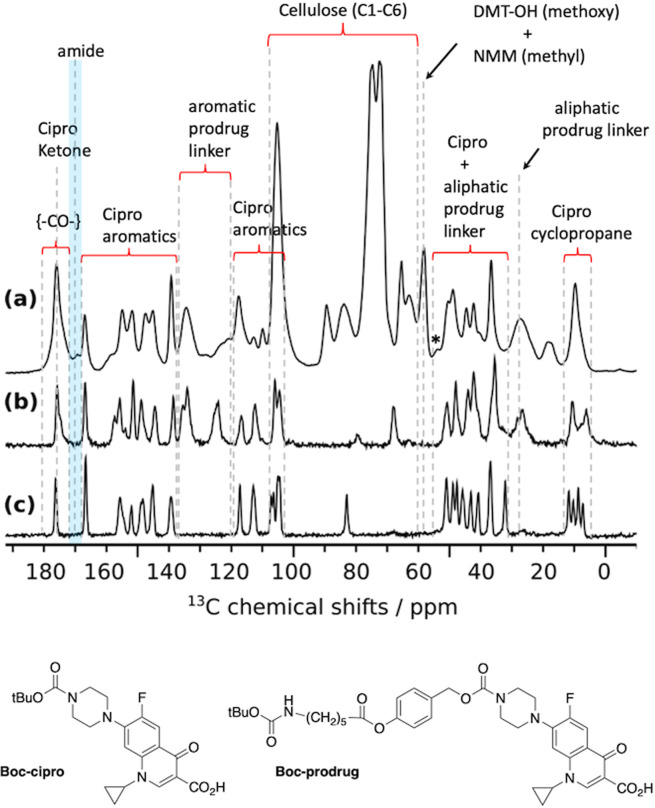
^13^C-CPMAS spectroscopy. **a** DNP-enhanced quantitative ^13^C-CPMAS spectrum of CNF-prodrug-pH6, acquired with the MultiCP pulse sequence^79^. The DNP sample was prepared by impregnation with the radical solution. **b**, **c** Solid-state ^13^C-CPMAS spectra (without DNP) of **b** Boc-prodrug and **c** Boc-cipro. Both samples were pure powders. All three spectra were recorded at 100 K. Spinning sidebands are marked with asterisks. Chemical structures of Boc-prodrug and Boc-cipro are given at the bottom of the figure.

The cipro-prodrug considered here consists of 31 carbons (see Fig. 1), which is about twice the number of carbons encountered in the dansyl-amine case. Nevertheless, thanks to the large sensitivity gain afforded by DNP, it is possible to extract atomic-scale information about the CNF surface species within a couple of hours of experimental time only. This is illustrated in Fig. 5a with the DNP-enhanced ^13^C-CPMAS spectrum of CNF-prodrug-pH6. Globally, this spectrum reveals a surprisingly large amount of drug in the sample, compared to what was observed in the dansyl-amine case. This strongly suggests the presence of prodrug adsorption. As spectral crowding makes the resonance assignment of this spectrum quite challenging, further solid-state NMR spectra of the Boc-protected prodrug (Boc-prodrug, Fig. 5b) and the Boc-protected ciprofloxacin (Boc-cipro, Fig. 5c) were recorded. The tertiary carbon of the Boc groups appears at around 80 ppm in both spectra. The resonances around 10 ppm are characteristics of the cyclopropyl ring and are present in all spectra. The same applies for other resonances from the cipro moiety: aromatic carbons and carbamate from 105 to 156 ppm, COOH at 166 ppm, and ketone resonance at 177 ppm. This confirms that the cipro moiety is present in CNF-prodrug-pH6. Furthermore, resonances at 125 and 135 ppm only appear in Fig. 5a, b, and can thus be assigned to the aromatic carbons of the prodrug linker. Overall, all resonances from the cipro and linker moieties in the Boc-prodrug spectrum (Fig. 5b) are also present in the CNF-prodrug-pH6 spectrum (Fig. 5a). The later contains as well the expected resonances from TEMPO-oxidized cellulose but also at least three additional signals at 17, 58 and 169.5 ppm. The signal at 58 ppm is consistent with the methoxy groups of DMT-OH, as well as two of the CH_2_ groups from *N*-methylmorpholine (NMM). Both DMT-OH and NMM are side-products from the DMTMM reaction (see Fig. S3). The other two CH_2_ resonances from NMM are expected around 67 ppm but cannot be observed because of spectral overlap with the resonances of cellulose C6. The methyl group from NMM is expected around 46 ppm and thus overlap with the aliphatic cipro-prodrug carbons. Aromatic carbons of DMT-OH are likely to overlap with cipro aromatic resonances. The signal at 17 ppm is unexpected and is attributed to a side-reaction product involving the cleavage of the prodrug linker (discussed in the following sections). The resonance at 169.5 ppm, which is only present in Fig. 5a, has been assigned to the amide bond resulting from the covalent grafting of the amine-terminated prodrug on CNF-t. A detailed analysis of the carbonyl region reveals after deconvolution (Fig. S4) that the amount of bounded prodrug represents about 3.0 ± 0.6% of the CNF glucose units. This is nearly one order of magnitude smaller than the total amount of prodrug in the sample, estimated at 30 ± 6% (obtained from deconvolution and integration, with respect to C4, of the ciprofloxacin resonances between 145 and 155 ppm). The high level of adsorption of the cipro-prodrug onto the CNF surface (in contrast to the dansyl-amine case) could be explained by its higher hydrophobicity and H-bonding capabilities. Another interesting difference with the dansyl-amine and benzylamine cases, is the presence of DMTMM and DMTMM by-products in the CNF-prodrug-pH6, which were almost absent in CNF-dansyl/DMTMM and CNF-^15^*N*-benzylamine. This is probably related to the large amount of prodrug (adsorbed and bonded) that can help trapping by-products on the CNF surface. It also shows that washings at neutral pH are not able to efficiently remove adsorbed chemical species (starting compounds or by-products).

### pH-controlled washing investigated by ^15^N and ^13^C DNP-enhanced ssNMR

**Fig. 6:**
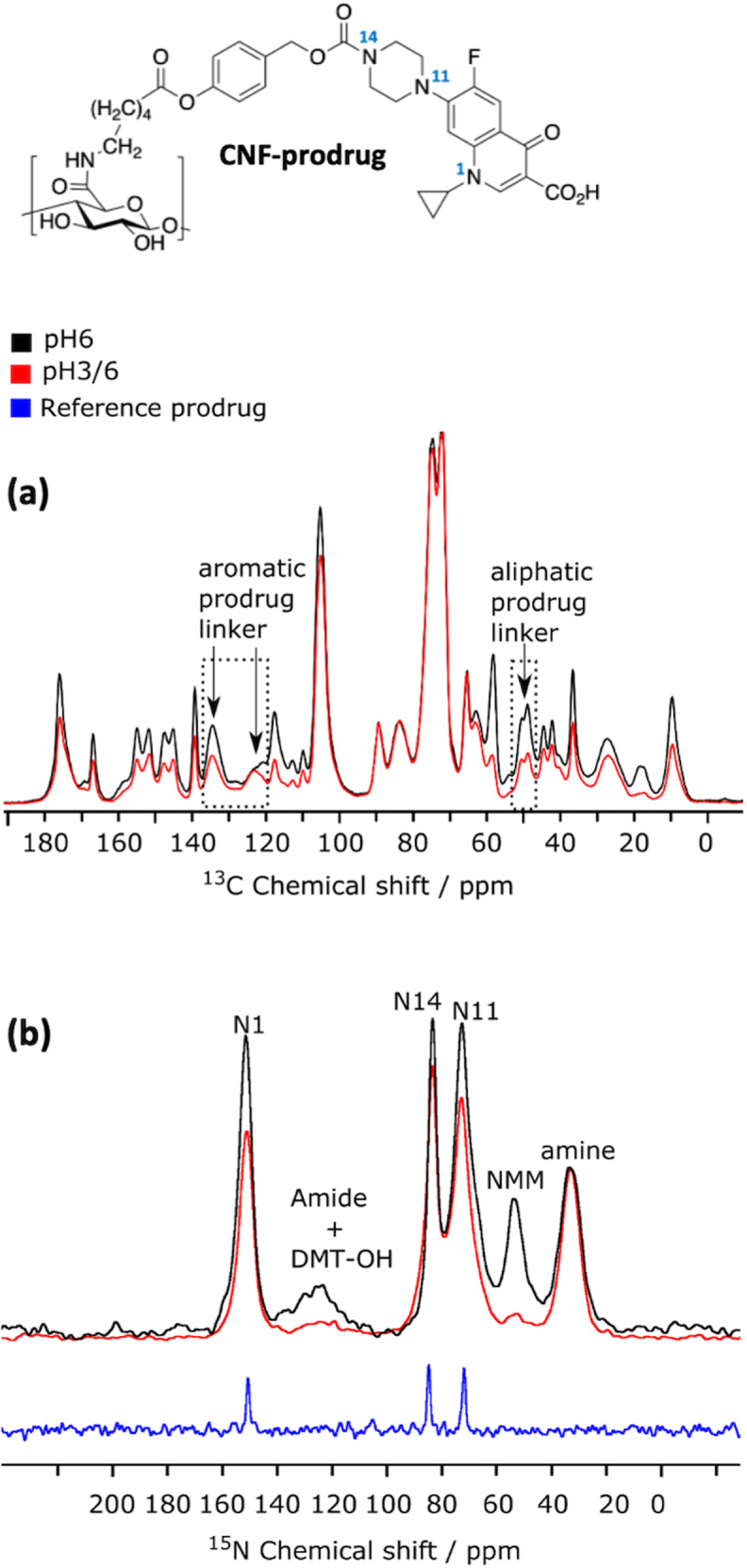
DNP-enhanced ^13^C and ^15^N-CPMAS spectroscopy. **a** DNP-enhanced quantitative ^13^C-CPMAS spectra of CNF-prodrug-pH3/6 (in red) and CNF-prodrug-pH6 (in black), both acquired with the MultiCP pulse sequence^79^. The two spectra were scaled to match the cellulose signals. **b** DNP-enhanced ^15^N-CPMAS spectra of CNF-prodrug-pH3/6 (in red) and CNF-prodrug-pH6 (in black). The ^13^C CPMAS spectrum (without DNP) of the Boc-protected cipro (crystalline powder) is given as reference (in blue). Peak assignment based on comparison with the ^15^N spectrum of Boc-protected cipro and published data^80,81^ is given in the figure. The ^15^N spectra were scaled such that the N1 peak ratio of the two CNF spectra reflects the difference by a factor of 2/3 in prodrug loading between the two samples, as determined in (**a**). The CNF-prodrug chemical structure is given at the top of the fig. with numbering of the nitrogen atoms.

Figure 6a compares the quantitative 1D ^13^C spectra of the two CNF-prodrug samples, CNF-prodrug-pH3/6 and CNF-prodrug-pH6, which differ in the applied washing procedure. It is clear from that comparison that the total amount of prodrug loading is one and half times smaller in CNF-prodrug-pH3/6 than in CNF-prodrug-pH6, for which it was estimated at 30 ± 6% (see above). This result is not surprising since the use of an acidic pH is expected to protonate the COOH and the terminal amine group of the prodrug, limiting the possibility of ionic interaction. Furthermore, by comparing the relative resonance intensities of the signal at 58 ppm in Fig. 6a, one can deduce that washing at pH 3 leads to a better removal of DMTMM side-products by a factor of ~3. This point is further confirmed by the comparison of the DNP-enhanced ^15^N CPMAS spectra of both samples given in Fig. 6b. By comparing the relative contribution of DMTMM side-products (115-140 ppm and 50 ppm) with respect to N1 (at 157 ppm), one clearly sees the importance of the acidic washing steps to remove coupling agent derivatives. This point is in full agreement with the results obtained with the dansyl dye.

Furthermore, it is interesting to note that in both samples, the ^15^N amide resonance, expected at 125 ppm and unfortunately overlapping with the DMT-OH signal, is much smaller than the ^15^N amine resonance at 35 ppm. Even though resonance overlapping prevent any quantitative analysis, this is qualitatively consistent with the results obtained above from the ^13^C DNP-enhanced CPMAS spectra, which showed that amine adsorption is about one order of magnitude larger than grafting. The amount of drug loading can however not be readily extracted from ^15^N NMR data, since no comparison can be made with the resonances from CNF.

### Insight into base-catalyzed prodrug cleavage using DNP-enhanced solid-state NMR

**Fig. 7:**
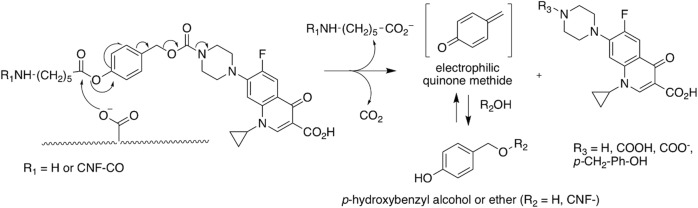
Expected mechanism for CNF-COO- base-catalyzed cleavage of the prodrug at neutral pH. The carboxylate present at the surface of CNFs can catalyze the ester bond cleavage and the release of ciprofloxacin.

The analysis above confirms that the coupling agent and derivatives are not efficiently washed away using neutral washing (pH 6). Nevertheless, a continuous release of the cipro-drug at pH 6 has been observed in the fluorescence experiments reported above. This is an interesting observation since such pH conditions are not expected to favor, a priori, acidic, or basic ester-bound cleavage. To explain this unexpected result, we propose an original mechanism where carboxylate anions present on the CNF-t surface at pH 6 catalyze the ester bound cleavage. A possible mechanism for this base-catalyzed reaction, which results in the release of ciprofloxacin and other chemical species, is depicted in Fig. 7. This release mechanism at neutral pH does not follow classical acid-base ester catalysis but bears some similarity with intramolecular carboxylate-catalyzed hydrolysis of esters^69^. It is worth pointing out that, to the best of our knowledge, such mechanism has never been reported except in the work of Carlsson et al.^70^, where they observed aspirin degradation in presence of surface-charged TEMPO-oxidized nanocellulose. We used DNP-enhanced ssNMR to check if we can detect this ester hydrolysis at neutral pH. This is far from trivial since it is not clear whether the released ciprofloxacin is washed away or partly adsorbed on the CNF surface. It is also worth pointing out that one cannot rule out that this mechanism starts already during the amidation reaction since it occurs at neutral pH.

While comparing DNP-enhanced quantitative ^13^C spectra of CNF-prodrug-pH6 and CNF-prodrug-pH3/6, we found that the ratio of signals at 125 and 135 ppm (highlighted by arrows in Fig. 6a) was different in the two samples (pH3/6 vs pH6). These two signals belong to aromatic carbons from the linker located between the ester and carbamate bonds (Fig. 6). Such variation in signal ratio from the aromatic linker can be explained by a chemical modification of the linker, like for instance a cleavage of prodrug linker. The latter is also consistent with the variation in ^13^C signal intensities observed at 50 ppm, which corresponds to some of the aliphatic CH_2_ groups of the linker (indicated by an arrow in Fig. 6a).

To further investigate this phenomenon, we compared the DNP-enhanced ^15^N CPMAS spectra of the two samples (Fig. 6b). The prodrug loaded on the CNF surface contains three nitrogen atoms N_cipro_ from the ciprofloxacin moiety (labeled N1, N11, and N14) as well as an amine/amide nitrogen (for adsorbed/grafted prodrug). Since the amine is one order of magnitude larger than the amide, one can use the amine to N_cipro_ signal ratio to qualitatively check whether the prodrug linker is intact. Although the ^15^N CPMAS experiment is not quantitative, it can still be used to compare the relative ratio of ^15^N resonances from two datasets (CNF-prodrug-pH3/6 *vs* CNF-prodrug-pH6) recorded with the same experimental parameters. According to the ^13^C NMR analysis, the amount of prodrug is x1.5 higher in the CNF-prodrug-pH6 sample. ^15^N data were therefore scaled accordingly in Fig. 6b to set the ratio of N1 signals to 1.5. With this scaling, the relative intensity of the amine signal in both samples is roughly in a 1:1 and not a 1:1.5 ratio, which is consistent with the presence of prodrug cleavage. In addition, the N_cipro_ to N_amine_ ratio is significantly higher in the CNF-prodrug-pH6 than in CNF-prodrug-pH3/6. This suggests that part of the ciprofloxacin released through prodrug cleavage ends up adsorbed on the CNF surface. Based on the above analysis, one can estimate that there is about a 1:2 ratio of ciprofloxacin:prodrug adsorbed on the CNF surface. We believe it is highly unlikely that the cleaved ciprofloxacin is bonded to CNF, yet it is difficult to confirm that unambiguously. In summary, for the CNF-prodrug-pH6 sample, we can estimate the amount of prodrug (adsorbed or covalently bounded) to ~20% ± 6% and of ciprofloxacin loaded on the CNF surface to ~10% ± 6%, both with respect to the number of CNF glucose units.

It should be noted that there might be other mechanisms of base-catalyzed cleavage similar to that shown in Fig. 7. Nonetheless, the common feature of all these mechanisms must be the cleavage of the linker in the prodrug and release of ciprofloxacin.

### Understanding prodrug adsorption on CNFs through quantitative structure-activity relationship (QSAR) and DNP-enhanced ssNMR

Several studies have notably discussed the mode of binding of cationic dyes onto negatively charged cellulose materials^71–73^. These efforts point to the importance of the electrostatic interactions, and their increase with the pH through the modification of the adsorbent surface charge and the ionization degree of the dye. Electrostatic interactions can occur if the cationic dye comes close enough to the anionic sites present on the surface of cellulose-based adsorbent. Liu et al.^72^ proposed that the positively heterocyclic dye migrating from the aqueous solution onto the surface of the material could be adsorbed through Van der Waals interaction. Formation of hydrogen bonding between the drug and the surface have also a positive role in the adsorption process.

To rationalize the high level of adsorption of cipro-prodrug onto CNFs compared to the dansyl-amine case on one side, and the pH-dependent washing efficiency discussed above on the other side, we analyzed the predicted quantitative structure-activity relationship (QSAR) of these two molecules, as well as of the reactants and reagents involved in the amidation reaction (Table S1).

**Fig. 8:**
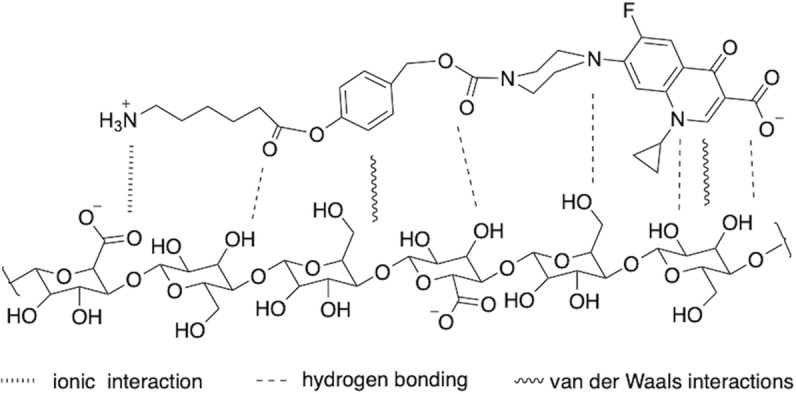
Adsorption of the ciprofloxacin-prodrug onto CNF-t. Possible stabilizing effects include ionic interaction, hydrogen bonding, and van der Waals interactions.

Dansyl-amine and cipro-prodrug show major differences in their QSAR characteristics. Based on the data reported in Table S1, one can expect the electrostatic interactions between the CNF surface and both molecules to be similar for acidic pH conditions but different for neutral pH. Indeed, both molecules are positively charged at acidic pH, whereas ciprofloxacin-drug exists as a zwitterion at neutral pH. In addition, the large number of possible hydrogen-bonding sites and the relatively large polar surface area of ciprofloxacin prodrug is expected to strengthen its adsorption onto the CNF surface as depicted in Fig. 8. Furthermore, negative LogD values for dansyl-amine can also explain why the extraction of adsorbed dansyl- amine is more efficient than for the lipophilic cipro-prodrug amine, especially in acidic water washing. Using the same argument, one can understand why the removal of DMTMM side products is favored in acidic water.

## Conclusion

The results reported herein highlight the critical importance of extracting atomic-scale information related to the surface chemistry of heterogeneous reactions. This is illustrated in the case of an amidation reaction performed on tempo-oxidized CNFs. In particular, we were able to discuss the advantages of using DMTMM over EDC/NHS as coupling agent, the difficulty of controlling covalent grafting *versus* adsorption for large hydrophobic prodrugs, and the impact of streamline washing procedures. Indeed, unlike for the dansyl-amine case where covalently bounded dansyl chromophores are the only detectable species, ciprofloxacin prodrug is largely adsorbed, at a level of nearly one order of magnitude higher than covalent grafted prodrug. This suggests that adsorption of the starting reactants depend on structural factors, such as protonation state, hydrophobicity, hydrogen bonding ability, etc.

Moreover, the DNP-enhanced ^13^C and ^15^N ssNMR results allowed to explain the fluorescence experiments, by proving the efficiency of the acidic pH washing in removing species adsorbed on the CNF surface, but also by revealing an unexpected phenomenon at neutral pH leading to the cleavage of the prodrug linker. This type of information is essential to further develop the use of CNFs in the context of drug delivery. Based on the DNP-enhanced ssNMR analysis, we proposed a mechanism to explain this phenomenon as the result of a base-catalyzed cleavage of the ester group triggered by surface carboxylate ions. The final product can therefore end up with a mixture of adsorbed released ciprofloxacin and both the covalently bonded as well as adsorbed prodrug.

This work has highlighted the need to develop more robust strategies for the functionalization of prodrugs on CNFs. Future efforts to improve the coupling and washing procedures should clearly benefit from the use of DNP-enhanced ssNMR, which, as demonstrated in this work, can provide unique insight into surface chemistry. It will also be essential to study new cleavable prodrug linkers with the goal of promoting covalent binding rather than adsorption. We anticipate that DNP-enhanced ssNMR will be a key approach to streamlining such developments as it is uniquely suited to provide atomic-scale information able to rationalize the molecular structure-adsorption relationship on CNFs.

## Methods

The oxidized cellulose nanofibrils (CNF-t) suspensions were prepared by Centre Technique du Papier (CTP, Grenoble, France). A brief description can be found in our previous publication^21^. The batch of CNF-t used for this work had a molar mass of 1.4 mmol/g dry of acid and about 0.7 mmol/g dry of aldehyde.

*N*-(6-Aminohexyl)-5-dansylsulfonamide^74^, coupling agent *N*-(4,6-dimethoxy-1,3,5-triazin-2-yl)-4-methylmorpholinium chloride (DMTMM)^75^, Boc-cipro, and the linker 4-(Hydroxymethyl)phenyl-6-((tert-butoxycarbonyl)-amino)hexanoate were prepared following published procedures^18,76^. The chemical synthesis of cipro-prodrug is given in SI.

### Reaction of CNF-t with N-(6-Aminohexyl)-5-dansylsulfonamide using EDC/NHS as coupling agents

The CNF-t suspension (18.40 g, 0.286 mmol) was diluted in pH 4 aqueous HCl (30 mL) at rt and the mixture was stirred during 5 min. EDC (0.12 g, 0.57 mmol) was dissolved in pH 4 aqueous solution HCl, (10 mL), and then added to the CNF-t suspension and stirred during 5 min at rt. NHS (0.068 g, 0.57 mmol) dissolved in pH 4 aqueous HCl (10 mL) was then added to the solution, which was magnetically stirred for 30 min at rt. N-(6-aminohexyl)-5-dansylsulfonamide (0.21 g, 0.57 mmol) dissolved in aqueous pH 3 HCl solution (10 mL) was added to the suspension. The pH of the suspension was then adjusted to 6.5 under magnetic stirring using a 0.5 M aqueous NaOH solution. The mixture was magnetically stirred for 24 h at 37 °C.

The reaction medium was centrifuged and the modified CNF-t was treated by repeated washing/centrifugation cycles. The removal of dansyl containing products (both excess of reagent and water-soluble by-products) was monitored by fluorescence spectra of the supernatant obtained after each cycle. A total of 5 centrifugation/dispersion cycles at pH 3 (HCl/H_2_O) were needed to remove all fluorescent product. The suspension was then treated by 3 or 4 centrifugation/dispersion cycles in deionized water to reach neutrality. The absence of N-(6-aminohexyl)-5-dansylsulfonamide in the last aqueous phase was controlled by HPLC analysis (UV-Vis detection). After freezing and lyophilization, 0.147 g of solid was obtained.

The –COOH concentration was estimated by conductometry. An average value of 1.5 mmol/g was obtained, in the same range as for the starting CNF-t. The aqueous phases were grouped and basified, before extraction of the unreacted amine in CH_2_Cl_2_. After drying in magnesium sulfate, the organic phase was evaporated to dryness to afford 0.156 g of starting N-(6-aminohexyl)-5-dansylsulfonamide, indicating that 0.05 g (~24%) of the reactant has been adsorbed or bound on CNF-t.

### Reaction of CNF-t with N-(6-Aminohexyl)-5-dansylsulfonamide using DMTMM as coupling agent

The CNF-t suspension (18.35 g, 0.288 mmol, 0.24 g of dry CNF-t) was diluted in distilled water (30 mL). The DMTMM (0.160 g, 0.57 mmol) dissolved in distilled water (10 mL) was added to the CNF-t suspension. The solution was magnetically stirred for 30 min at rt.

N-(6-aminohexyl)-5-dansylsulfonamide (0.21 g, 0.57 mmol) dissolved in aqueous pH 3 HCl solution (10 mL) was added to the suspension. The pH of the resulting suspension was adjusted to pH 6.5. After 4 h of reaction at 37 °C, the reaction medium was centrifuged and the modified CNF-t was treated by repeated washing/centrifugation cycles as described above. The modified CNF-t residue was frozen at −39.5 °C and lyophilized to give 0.143 g of solid.

The –COOH was estimated by conductometry. An average value of 1.27 mmol/g was obtained. The aqueous phases were grouped and basified, before extraction of the unreacted amine in CH_2_Cl_2_. After drying in magnesium sulfate, the organic phase was evaporated to dryness to afford 0.16 g of starting N-(6-aminohexyl)-5-dansylsulfonamide, indicating that 0.05 g (24%) of the reactant has been adsorbed or bound on CNF-t.

### Functionalization of CNF-t with ^15^N-benzylamine using DMTMM

The CNF-t suspension (26.02 g, 0.406 mmol) was diluted in water (30 mL). DMTMM (0.277 g, 1 mmol) dissolved in water (10 mL) was added to the CNF-t suspension. The solution was magnetically stirred for 30 min at rt. ^15^N- benzylamine (110 µL, 1 mmol) and water (5 mL) were added to the suspension that was then magnetically stirred at 37 °C. After 4 h of reaction, purification of the mixture was performed by repeated washing/centrifugation cycles as previously described. The mixture was frozen (−39.5 °C) and lyophilized to yield 0.27 g of solid (79% of the starting dried CNF-t).

### Functionalization of CNF-t with ciprofloxacin prodrug using DMTMM

The reaction was performed twice in the same reaction conditions using, however, different conditions for product washing.

The CNF-t suspension (18.35 g, 0.35 mmol -COOH) was diluted in water (20 mL) and DMTMM (0.160 g, 0.57 mmol) dissolved in water (10 mL) was added to the CNF-t suspension. The solution was magnetically stirred for 30 min at 37 °C.

Ciprofloxacin prodrug (0.46 g, 0.57 mmol) dissolved in water (20 mL) was added to the suspension. After 4 h of reaction at 37 °C, the reaction medium was centrifuged and the modified CNF-t was treated by repeated washing/centrifugation cycles as described below. The presence of ciprofloxacin chromophore in the supernatant was checked by HPLC analysis (UV-Vis detection) after each washing.

The first batch was washed with pH 3 aqueous HCl as described above. After five acidic and four neutral washing/centrifugation cycles, traces of ciprofloxacin chromophore were still observed in the supernatant. The second batch was washed ten times with distilled water. A slow and continue desorption of ciprofloxacin chromophore was observed but no trace was seen on the HPLC chromatogram after the last washing.

### DNP-enhanced solid-state NMR

DNP samples of the different products were prepared by impregnation with a DNP matrix composed of 10 mM AsymPolPOK^54^ in D_2_O only. A glass forming solvent was not used on purpose to avoid any spectral overlap with solvent resonances. Amounts of product and radical solution were, respectively, of 25 mg and 25 µLfor CNF-dansyl/EDC-NHS, 25 mg and 30 µL for CNF-dansyl/DMTMM, 20 mg and 40 µL for both CNF-prodrug/pH3/6 and CNF-prodrug/pH6, 20 mg and 30 µL for CNF-^15^N-benzylamine. Each impregnated DNP sample was then fully packed into a 3.2 mm outer-diameter sapphire rotor.

DNP experiments were performed at CEA/Grenoble Alpes University on a Bruker Avance III 400 MHz (^1^H resonance) DNP-NMR spectrometer equipped with a 263 GHz gyrotron for microwave irradiation, a corrugated transmission line and a low temperature 3.2 mm MAS probe used in double-resonance mode^49^. All experiments were performed at a sample temperature of 100 K and a magic-angle spinning (MAS) frequency of 12 kHz, unless stated otherwise. Radio frequency (rf) field strengths were set to 50 kHz on the ^13^C and ^15^N channels, and to 100 kHz on the ^1^H channel. The inter-scan delay was optimized according to the polarization build-up time of each sample, and set to 2.5 s for CNF-dansyl/EDC-NHS, 2.3 s for CNF-dansyl/DMTMM, 1.9 s for CNF-prodrug-pH3/6 and 2.0 s for CNF-prodrug-pH6, unless stated differently. The interscan delay (at rt) for the two reference samples Boc-protected-cipro and Boc-protected-prodrug was set to 5.0 s and 15.6 s, respectively.

For ^15^N CPMAS experiments, the CP contact time was set to 4 ms. A 50-to-100% ramp was used for the ^1^H CP spin-lock^77,78^. The CP decay time constants were measured and found to be in excess of 25 ms for all samples. 5888, 8196 and 1024 transients were accumulated for the ^15^N CPMAS spectra of CNF-prodrug-pH3, CNF-prodrug-pH6 and the Boc-protected-cipro, respectively.

For standard ^13^C CPMAS experiments, the CP contact time was set to 2 ms with a 50-to-100% ramp on the ^1^H channel. 2304, 1024, 150, and 256 transients were accumulated for the ^13^C CPMAS spectra of CNF-dansyl/DMTMM, CNF-dansyl/EDC-NHS, Boc-protected-prodrug, and Boc-protected-cipro, respectively. For the 1D z-filtered {^15^N}-^13^C TEDOR experiment on CNF-^15^N-benzylamine, the interscan delay, mixing time, and z-filter duration were set to 2.2 s, 2 ms, and 2.5 ms, respectively. 256 transients were accumulated at MAS frequency of 8 kHz. For the quantitative CP experiments using the Multi-CP^79^ pulse sequence, the CP contact time was set to 1 ms with a 70-to-100% ramp on the ^1^H channel, while the CP decay time constants were measured to be 25–35 ms. This ensures that the relative losses during each CP block are minimal. The total CP time is set to 10 ms (10 blocks of 1 ms) which is much longer than the CP build-up time (even for non-protonated carbon resonances). Given that the ^1^H and ^13^C longitudinal build-up times under DNP conditions (corresponding to longitudinal relaxation in non-DNP conditions) are <3 s and >35 s, respectively, we used an inter-contact delay of <2 s, ensuring significant ^1^H repolarization and negligible ^13^C polarization losses. The inter-scan delay and the delay between each CP block were, respectively, set to 1.9 s and 1.5 s for CNF-prodrug-pH3, and 2.0 s and 1.6 s for CNF-prodrug-pH6. 64 transients were accumulated for each spectrum. This ensures that the relative intensities of the ^13^C resonances can be compared.

All experiments were processed and analyzed using Bruker Topspin 3.2 software, including peak deconvolution and integration. ^13^C peak intensities are discussed relatively to cellulose C4 intensity, set to 100, as a measure of the glucose unit magnetization. The signal-to-noise of DNP-enhanced ^13^C MultiCP NMR spectra was above 10 for the smallest carbon signals and above 500 for cellulose C4. The overall error in the intensities obtained by deconvolution and integration was estimated at 10% for the MultiCP experiments, 5% for standard CP experiments on CNF-t and CNF-dansyl/EDC-NHS and 10% for the CP experiment on CNF-dansyl/DMTMM. Higher deconvolution error in CNF-dansyl/DMTMM is due to the poor signal-to-noise ratio of the experiment. ^13^C NMR chemical shift predictions were performed with Mnova NMR Predict from Mestrelab Research to help assignment of the spectra.

## Supplementary information

Supplementary Information

Description of Additional Supplementary Files

Supplementary Data 1

## Data Availability

Solution-state NMR spectra can be found in Supplementary Data 1. Other data that support the findings of this study are available from the corresponding author upon reasonable request.
