# Supplementary material for: Optimizing chemistry at the surface of prodrug-loaded cellulose nanofibrils with MAS-DNP
Source: Commun Chem. 2023 Mar 28;6:58. doi: 10.1038/s42004-023-00852-2 (PMC10049993; doi:10.1038/s42004-023-00852-2)
Supplement: Supplementary file 1 — Supplementary Information [file 42004_2023_852_MOESM1_ESM.pdf]

Supplementary Information

Optimizing chemistry at the surface of prodrug-loaded cellulose nanofibrils with MAS-DNP

Akshay Kumar,[1] Bastien Watbled,[2] Isabelle Baussanne,*[2] Sabine Hediger,[1] Martine Demeunynck,[2] Gaël De Paëpe*[1]

[1]Univ. Grenoble Alpes, CEA, CNRS, IRIG-MEM, Grenoble, France,

[2]Univ. Grenoble Alpes, CNRS, DPM, Grenoble, France.

Correspondence and request for material should be addressed to I.B. (email: isabelle.Baussanne@univ-grenoble-alpes.fr) and G.D.P. (email: gael.depaepe@cea.fr)

Supplementary Methods

A. Prodrug synthesis and characterization.

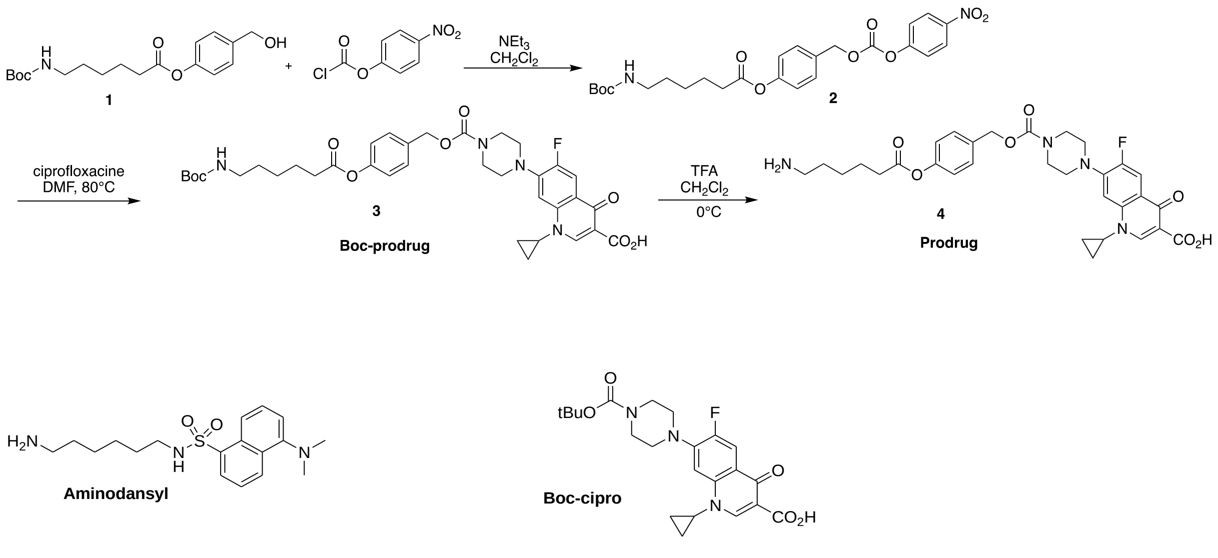

Scheme S1. Synthesis of the ciprofloxacine prodrug and structures of the two reference compounds, aminodansyl and Boc-cipro.

- Material and methods:

Solution-state NMR spectra were recorded at 298 K in 5 mm tubes on a Bruker AC 400 MHz spectrometer (NMR facility, PCN-ICMG, Grenoble). Chemical shifts (δ) are reported in parts per million (ppm) from low to high field and referenced to residual non-deuterated solvent relative to Me_4Si. Standard abbreviations for multiplicity were used as follows: s = singlet; d = doublet; t = triplet; m = multiplet. High-resolution mass sectrometry (HRMS) was carried out on a Bruker UHR-Q-TOF MaXis-ETD (Time of Flight) mass spectrometer using ElectroSpray Ionisation (ESI) in Institut de Chimie Organique et Analytique (CBM-ICOA) in Orleans (France).

N-Hydroxysuccinimide (NHS, CAS: 6066-82-6), N-(3-Dimethylaminopropyl)-N-ethylcarbodiimide hydrochloride (EDC, CAS: 25952-53-8), 2-Chloro-4, 6-dimethoxy-1, 3, 5-triazine (CDMT, CAS: 3140-73-6), 4-methylmorpholine (NMM, CAS: 109-02-4), Benzylamine-^{15}N (CAS: 42927-57-1), Sodium Hydroxide (NaOH, CAS: 1310-73-2), Hydrogen chloride (HCl, CAS: 7647-01-0), Tetrahydrofuran (THF, CAS: 109-99-9) were purchased from Sigma Aldrich or Acros Organics and used without further purification. The N-(6-Aminohexyl)-5-dansylsulfonamide (**Aminodansyl),**[1] the coupling reagent N-(4,6-dimethoxy-1,3,5-triazin-2-yl)-4-methylmorpholinium chloride (DMTMM),[2] the linker 4-(hydroxymethyl)phenyl-6-((tert-butoxycarbonyl)-amino)hexanoate, and **Boc-cipro** were prepared following published procedures.[3,4]

- Synthesis of the ciprofloxacine derived prodrug:

4-({[(4-nitrophenoxy)carbonyl]oxy}methyl)phenyl 6-{[(tert-butoxy)carbonyl]-amino}hexanoate (2)

To a solution of 4-(hydroxymethyl)phenyl 6-{[(tert-butoxy)carbonyl]amino}hexanoate[r3] **1** (1 g, 2.9 mmol**)** in anhydrous CH_2Cl_2 (2O mL) were added NEt_3 (500µL, 3.5 mmol) and *p*-nitrophenyl chloroformate (610 mg, 3 mmol). The yellow reaction mixture was stirred at r.t. for 2.5 h. The reaction mixture was then diluted with CH_2Cl_2 (60 ml) and washed with saturated aq. $NaHCO_3$ (3 x 60 mL), water (60 mL), HCl 0.5 M (60 mL) and then brine (60 mL). The organic phase was dried over $MgSO_4$ and filtered. Removal of the solvents under vacuum gave a yellow oil, which was purified by flash chromatography on silica gel with CH_2Cl_2/MeOH : 100/0 to 98/2 as eluent to give the compound **2** as an amorphous solid (1.23 g, 85 %).^{1}H NMR (400MHz, $CDCl_3$) δ 15.0 (s, 1H), 8.83, (s, 1H), 8.10 (d, 1H, *J*=12.8 Hz), 7.45 (d, 2H, *J*=8.6 Hz), 7.42 (d, 1H, *J*=7.1 Hz), 7.13 (d, 2H, *J*=8.6 Hz), 5.2 (s, 2H), 4.59 (br s, 1H), 3.79 (m, 4H), 3.59 (m, 1H), 3.35 (m, 4H), 3.19 (m, 2H), 2.62 (t, 2H, *J*=7.4 Hz), 1.82 (m, 2H), 1.64-1.56 (m, 4H), 1.49 (s, 9H), 1.49-1.44 (m, 2H), 1.25 (m,2H); ^{13}C NMR (100 MHz, $CDCl_3$) δ 172.1, 167.0, 155.0, 150.7, 147.6, 139.1, 133.9, 129.5, 121.8, 112.9, 112.6, 108.4, 105.2, 66.9, 49.7, 35.3, 34.3, 29.8, 28.5, 26.3, 24.6, 8.3.

7-{4-[({4-[(6-{[(tert-butoxy)carbonyl]amino}hexanoyl)oxy]phenyl}methoxy)carbonyl]-piperazin-1-yl}-1-cyclopropyl-6-fluoro-4-oxo-1,4-dihydroquinoline-3-carboxylic acid: Boc-Prodrug (3)

Compound **2** (358 mg, 0.71 mmol) was dissolved in dry DMF (6 mL). Ciprofloxacine (260 mg, 0.78 mmol) was added to the solution. The reacting mixture was stirred and warmed at 80°C for 3 h. The solution was concentrated under vacuum. The solid was washed with Et_2O 4 times to give pure compound **3** as a white solid (470 mg, 95%).
^{1}H NMR (400MHz, $CDCl_3$) δ 8.27 (d, 2H, J=2.2 Hz), 7.46 (d, 2H, J=8.6 Hz), 7.36 (d, 2H, J=9.2 Hz), 7.12 (d, 2H, J=8.6 Hz), 5.28 (s, 2H), 4.55 (br s, 1H), 3.14 (m, 2H), 2.57 (t, 2H, J=7.6 Hz), 1.77 (m, 2H), 1.54 (m, 4H), 1.44 (s, 9H); ^{13}C NMR (100 MHz, $CDCl_3$) δ 172.0, 156.1, 155.6, 152.5, 151.4, 145.6, 131.8, 130.2, 125.4, 122.2, 121.9, 79.3, 70.4, 40.5, 34.4, 29.9, 28.6, 26.4, 24.7.

7-{4-[({4-[(6-aminohexanoyl)oxy]phenyl}methoxy)carbonyl]piperazin-1-yl}-1-cyclopropyl-6-fluoro-4-oxo-1,4-dihydroquinoline-3-carboxylic acid: Prodrug (4)

Compound **3** (470 mg) was dissolved at 0°C in CH_2Cl_2 (8 mL). TFA (4 mL) was added to the solution, which was stirred at 0°C for 1h. The solution was concentrated under vacuum and the TFA co-evaporated with methanol and then toluene 3 times each. The deprotected compound **4** was obtained as a white solid (540 mg, quant. yield).^{1}H NMR (400MHz, CD_3OD) δ 8.82, (s, 1H), 7.97 (d, 1H, J=13.0 Hz), 7.61 (d, 1H, J=7.3 Hz), 7.44 (d, 2H, J=8.5 Hz), 7.10 (d, 2H, J=8.5 Hz), 5.18 (s, 2H), 3.74 (br m, 5H), 3.37 (br m, 4H), 2.95 (t, 2H, J=7.4 Hz), 2.64 (t, 2H, J=7.3 Hz), 1.82-1.67 (m, 4H), 1.52 (m, 2H), 1.40 (m, 2H), 1.21 (br m,2H); ^{13}C NMR (100 MHz, DMSO-d6) δ 176.4, 171.7, 165.8, 158.4, 158.1, 154.4, 154.2, 151.7, 150.1, 148.1, 144.9, 139.1, 134.3, 129.0, 121.8, 118.8, 111.1, 110.9, 106.9, 106.8, 106.7, 65.9, 49.1, 46.4, 42.6, , 35.8, 33.2, 26.6, 25.1, 23.7, 7.5; HRMS (ESI) m/z: calcd. for $C_{31}H_{36}FN_4O_7$ $[M+H]^+$ 595.2563, obsd 595.2560 and calcd. for $C_{31}H_{37}N_4O_{Å}$ $[M+2H]^{2+}$ 298.1318, obsd 298.1317.

Solution-state NMR spectra can be found in Supplementary Data 1 file.

- QSAR calculations.

Table S1. Predicted properties of the starting reactants, reagents and their side-products.

Reactant/reagents	pKa	LogD at pH 3, pH 7	Polar surface area (Å^2) at pH 7	Molecular surface area area (Å^2) at pH 7	Donor/ acceptor sites at pH 7
Ciprofloxacin	5.5 and 10.2	-2.0, -1.3	72.9	440.9	D2/A9

Cipro-prodrug	5.5 and 10.2	0.0, 0.6	147.1	838	D1/A13
Dansyl-amine	10.2	-2.4, -0.3	77.0	570.6	D2/A5
Benzylamine	9.3	-2.3, -1.3	27.6	186.9	D3/A0
NHS	7.2	-1.3, -1.5	57.6	153.1	D1/A6
EDU*	9.3	-4.3, -2.7	45.5	341.1	D3/A2
DMT-OH	3.8	0.9, -2.0	72.3	201.5	D0/A8

The calculations have been performed using MarvinSketch 19.9 software (http://www.chemaxon.com); for LogD calculations, the Consensus option, built on the ChemAxon and Klopman1 models and the PhysProp database, was chosen.

*EDU = urea from EDC

B. Fluorescence analysis of the washing solutions of CNF-prodrug samples

The washing phases of each batch of CNF-prodrug were combined and then diluted to 1L in water. The presence of released ciprofloxacin chromophore was assayed by fluorescence spectroscopy. Note that the pH of the two samples are different, preventing any precise comparison.

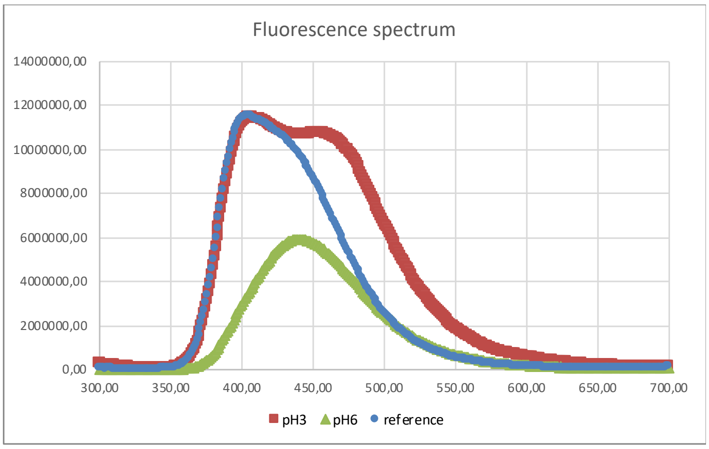

Figure S1. Fluorescence spectra of the combined washing phases of CNF-prodrug-pH3/6 (in red) and CNF-prodrug-pH6 (in green) obtained after washing at pH 3/pH 6 or pH 6 alone, respectively. The ciprofloxacin prodrug (conc. $4x10^{-5}$ mmol/L), spectrum in blue, is used as reference.[5]

Note: Due to presence of amine in the molecule, the fluorescence of ciprofloxacin is highly dependent on the pH. Nevertheless, the emission band of CNF-prodrug-pH3/6 is close to the one of ciprofloxacin at near neutral pH.[5]

C. Deconvolution of CNF-dansyl/EDC CPMAS spectra (110 to 170 ppm):

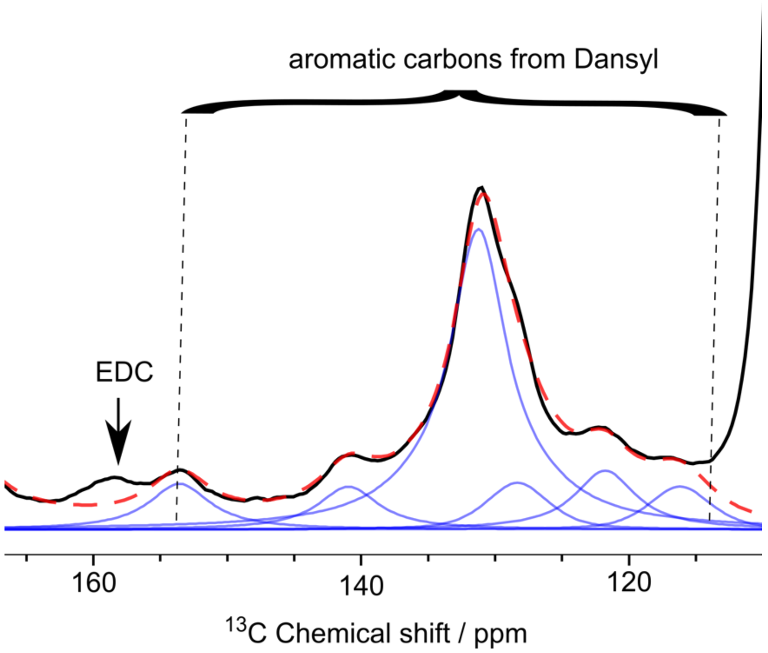

Figure S2. Deconvolution (in blue) of DNP enhanced ^{1}H-^{13}C CPMAS NMR spectrum of CNF-dansyl/EDC (in black). The sum of the deconvolution components is given in dashed red. The number of aromatic carbons (10 in our case) contributing to the spectrum was estimated by integrating the different components of the deconvolution, which gives relative integrals of about 1:1:5:1:1:1 (from the left to the right of the spectrum).

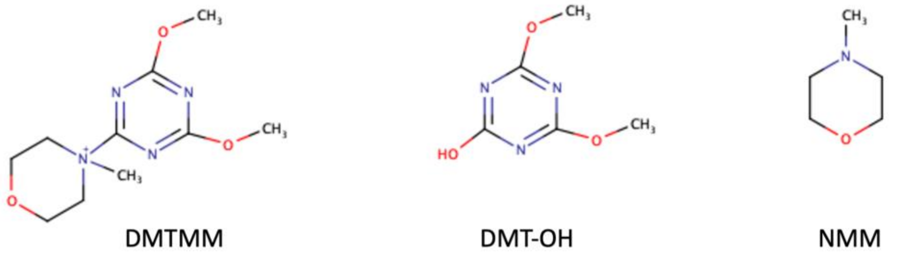

Figure S3. Chemical structure of DMTMM and its reaction side-products DMT-OH and NMM.

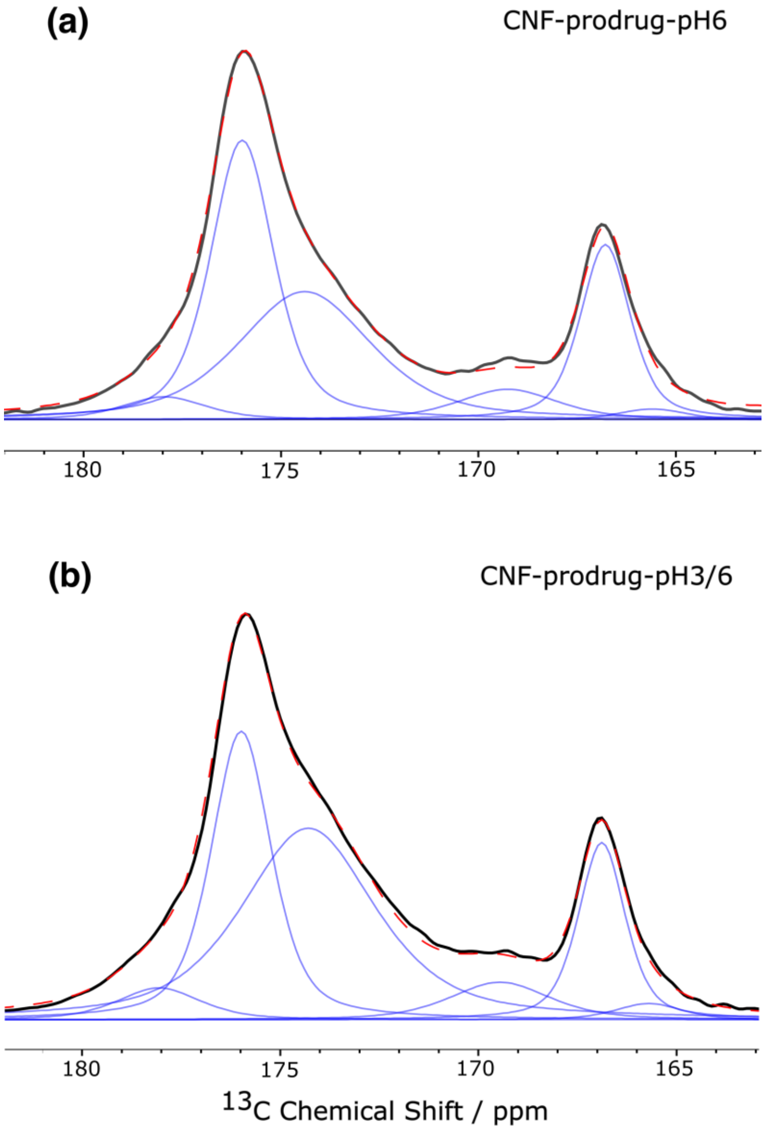

Figure S4. Signal deconvolution (in blue) of the region between 163 to 182 ppm in ^{13}C CPMAS spectra (in black) of (a) CNF-prodrug-pH6 and (b) CNF-prodrug-pH3/6. The sum of the deconvolution components is shown by dashed red lines. The two signals at 167 ppm and 176.5 ppm correspond to cipro signals (COOH and ketone) and have similar FWHH (full width at half height). The signal at 169.5 ppm corresponds to the amide resonance and has a FWHH identical to the one found in Fig 3. The signal at 174.5 ppm has a larger FWHH than the three others but corresponds to overlapping -COO- resonances, from the prodrug (ester carbon in the linker), DMTMM side products, and residual carboxylic groups from the CNF surface. This deconvolution together with integration over the different components has been used to estimate the amount of grafted prodrug.

Supplementary references:

[1] J. Wang, D.-T. Pham, T. W. Kee, S. N. Clafton, X. Guo, P. Clements, S. F. Lincoln, R. K. Prud'homme, C. J. Easton, *Macromolecules* **2011**, *44*, 9782–9791.

[2] S. A. Raw, *Tetrahedron Lett.* **2009**, *50*, 946–948.

[3] D. Bliman, M. Demeunynck, P. Leblond, S. Meignan, I. Baussane, S. Fort, *Bioconjug. Chem.* **2018**, *29*, 2370–2381.

[4] D. Das, S. Srinivasan, A. M. Kelly, D. Y. Chiu, B. K. Daugherty, D. M. Ratner, P. S. Stayton, A. J. Convertine, *Polym. Chem.* **2016**, *7*, 826–837.

[5] M. A. Al-Omar, *Profiles Drug Subst. Excipients Relat. Methodol.* **2005**, *31*, 179–207.
