# Supplementary material for: Optimizing chemistry at the surface of prodrug-loaded cellulose nanofibrils with MAS-DNP
Source: Commun Chem. 2023 Mar 28;6:58. doi: 10.1038/s42004-023-00852-2 (PMC10049993; doi:10.1038/s42004-023-00852-2)
Supplement: Supplementary file 2 — Description of Additional Supplementary Files [file 42004_2023_852_MOESM2_ESM.pdf]

Description of Additional Supplementary File

File name: Supplementary Data 1

Description: Solution-state NMR spectra data.
