# Supplementary material for: Optimizing chemistry at the surface of prodrug-loaded cellulose nanofibrils with MAS-DNP
Source: Commun Chem. 2023 Mar 28;6:58. doi: 10.1038/s42004-023-00852-2 (PMC10049993; doi:10.1038/s42004-023-00852-2)

Compound 2

^{1}H NMR (400 MHz, CDCl3)

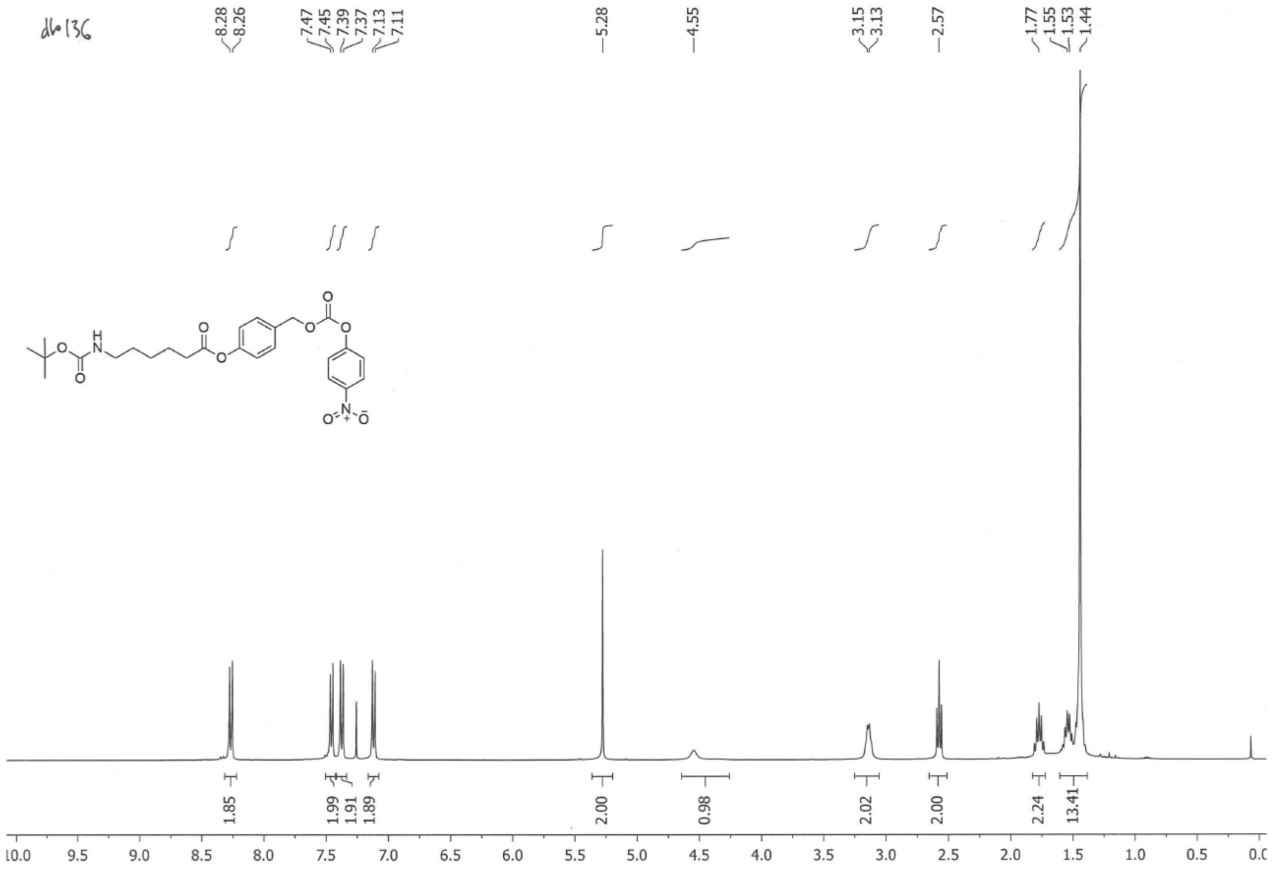

^{13}C NMR (100 MHz, CDCl3)

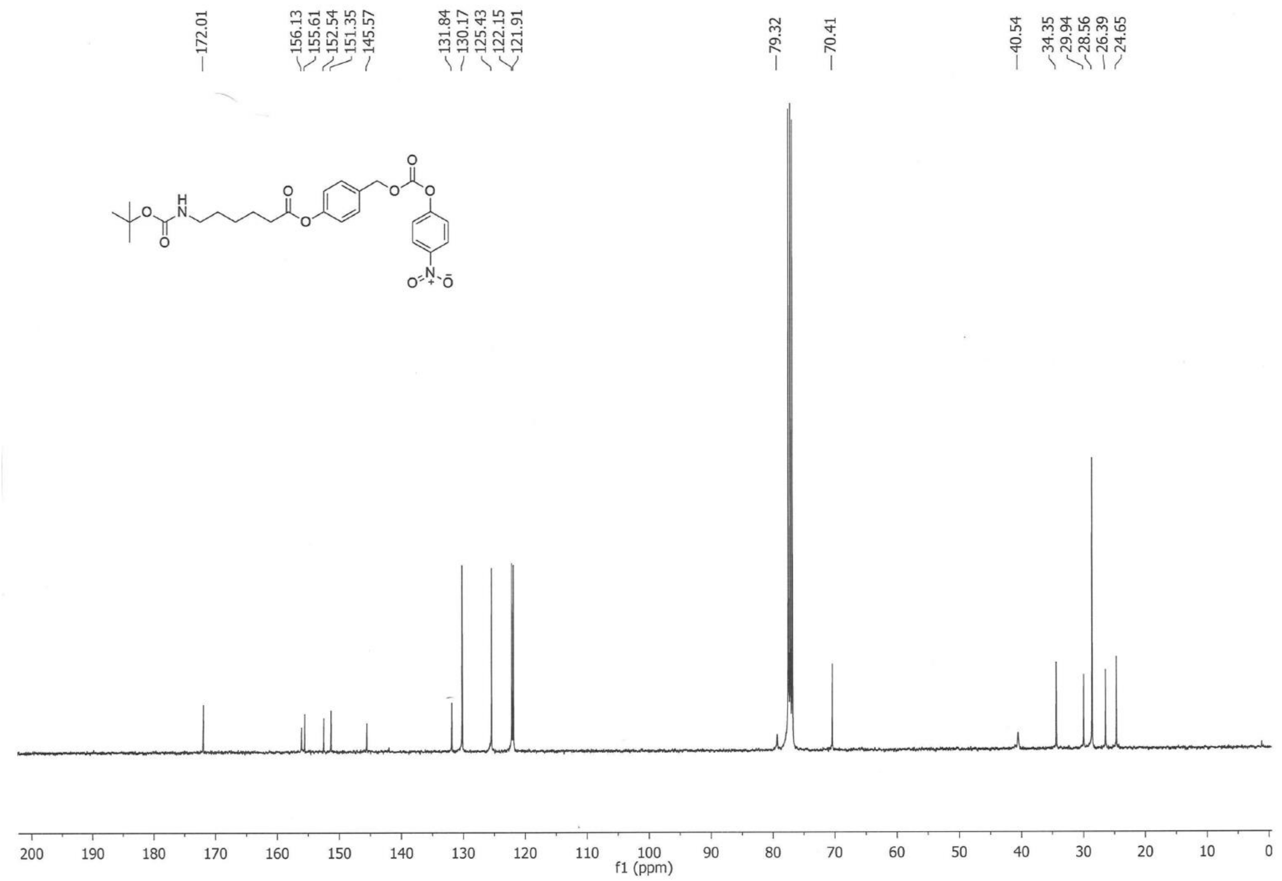

Boc-Prodrug 3

^{1}H NMR (400 MHz, CDCl3)

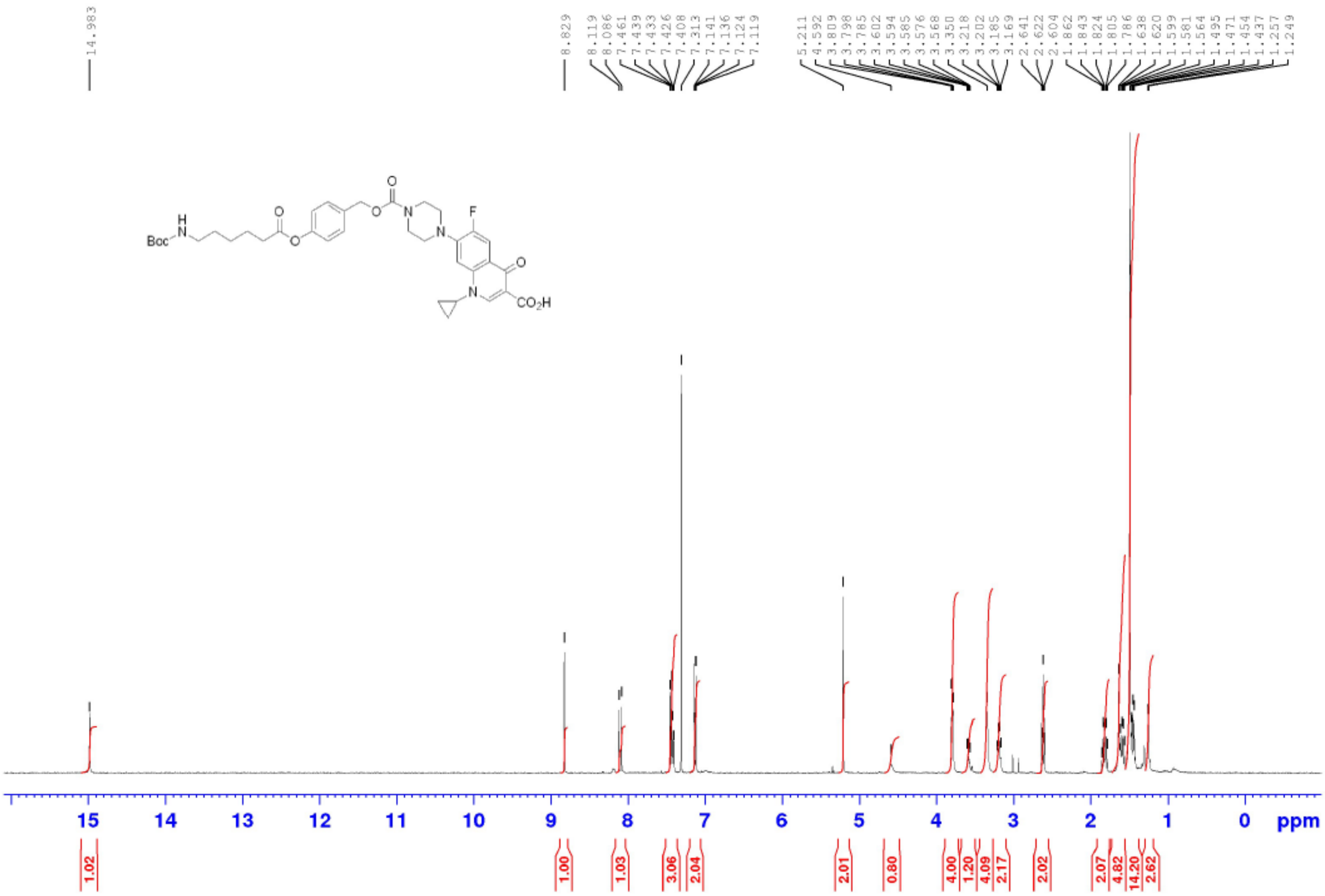

^{13}C NMR (100 MHz, CDCl3)

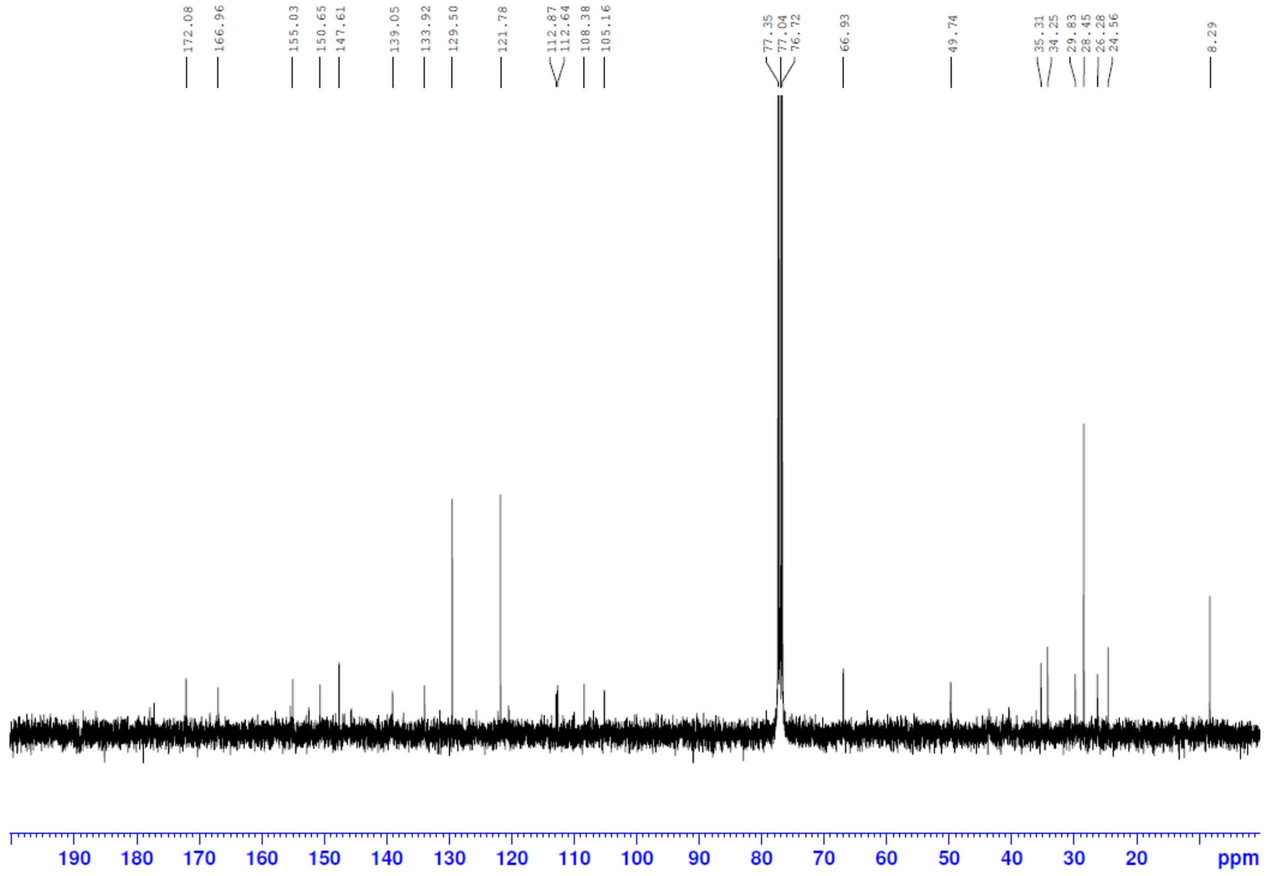

Prodrug 4

^{1}H NMR (400 MHz, CDOD)

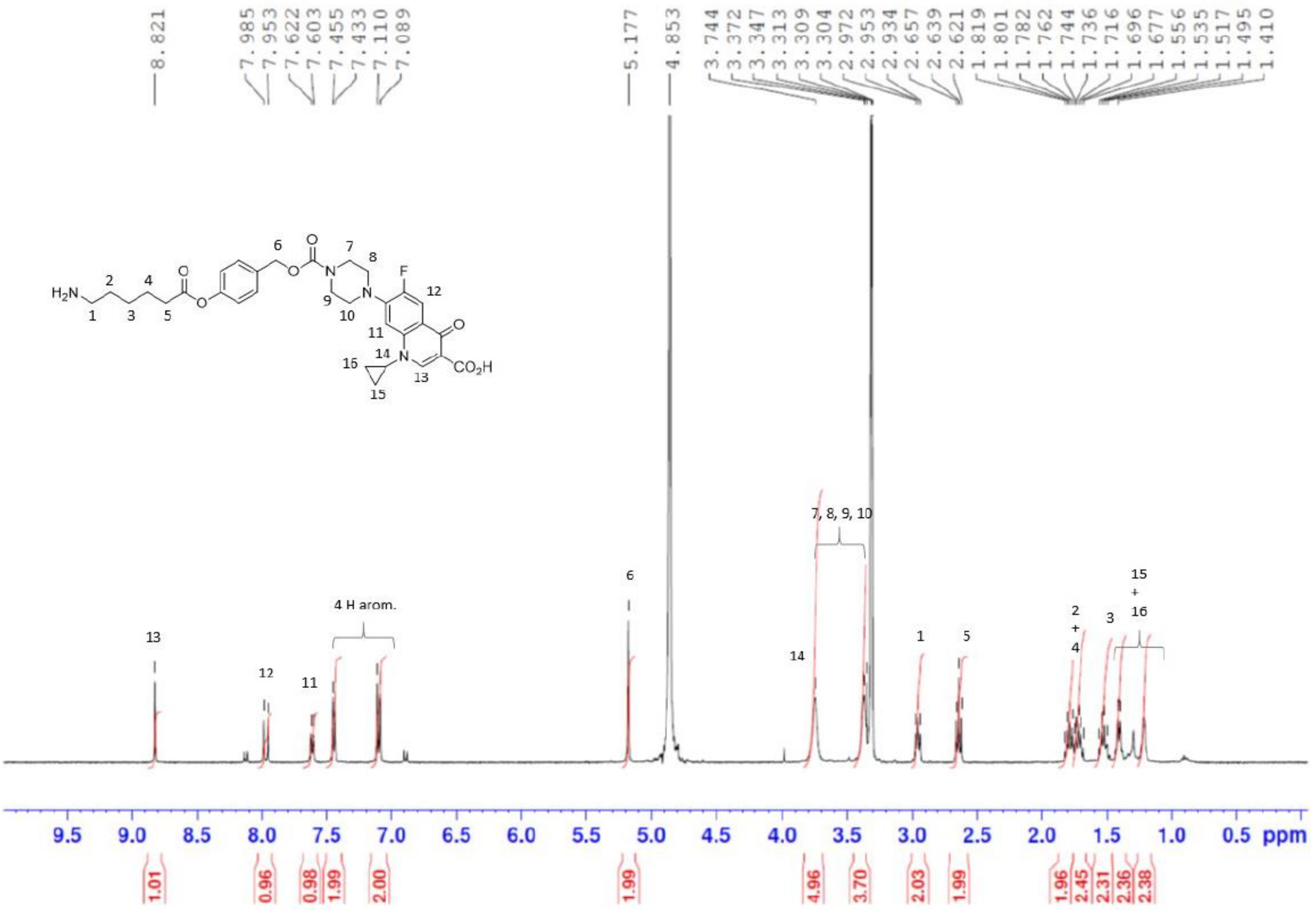

^{13}C NMR (13C{1H}-pulprog JMOD, 100 MHz, DMSO-d_6)

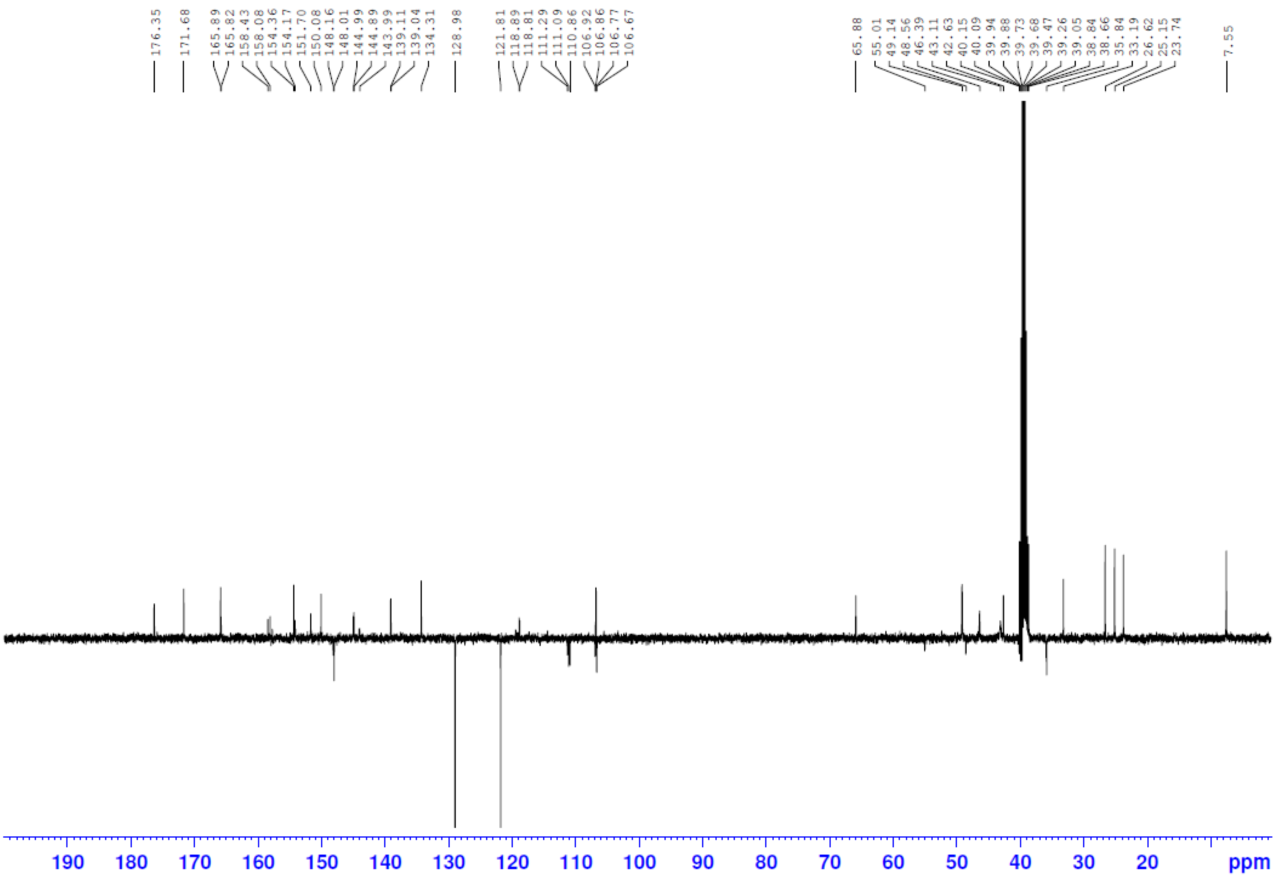

Aminodansyl

^{1}H NMR (400 MHz, CD_3Cl)

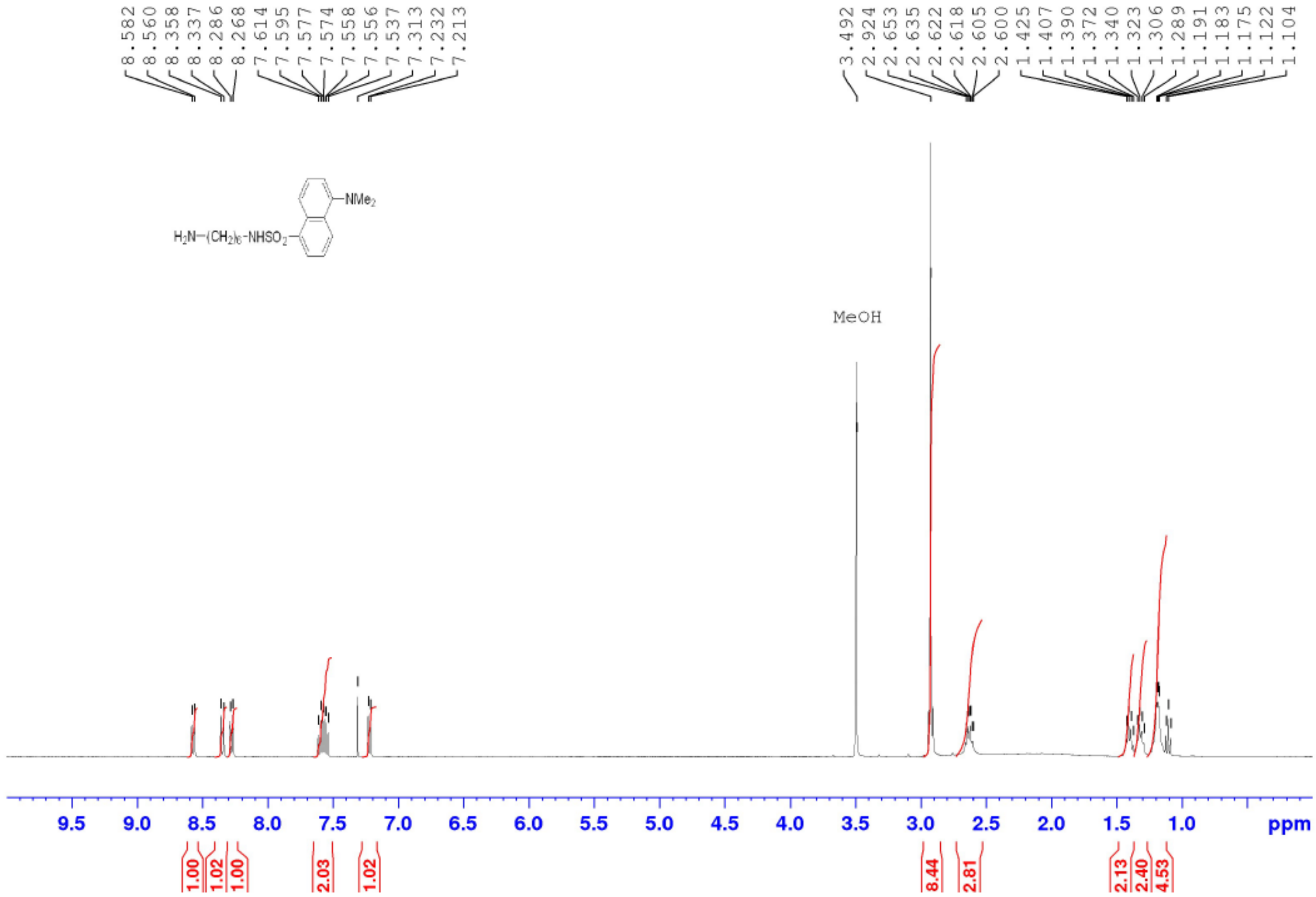

Boc-cipro

^{1}H NMR (400 MHz, CD_3Cl)

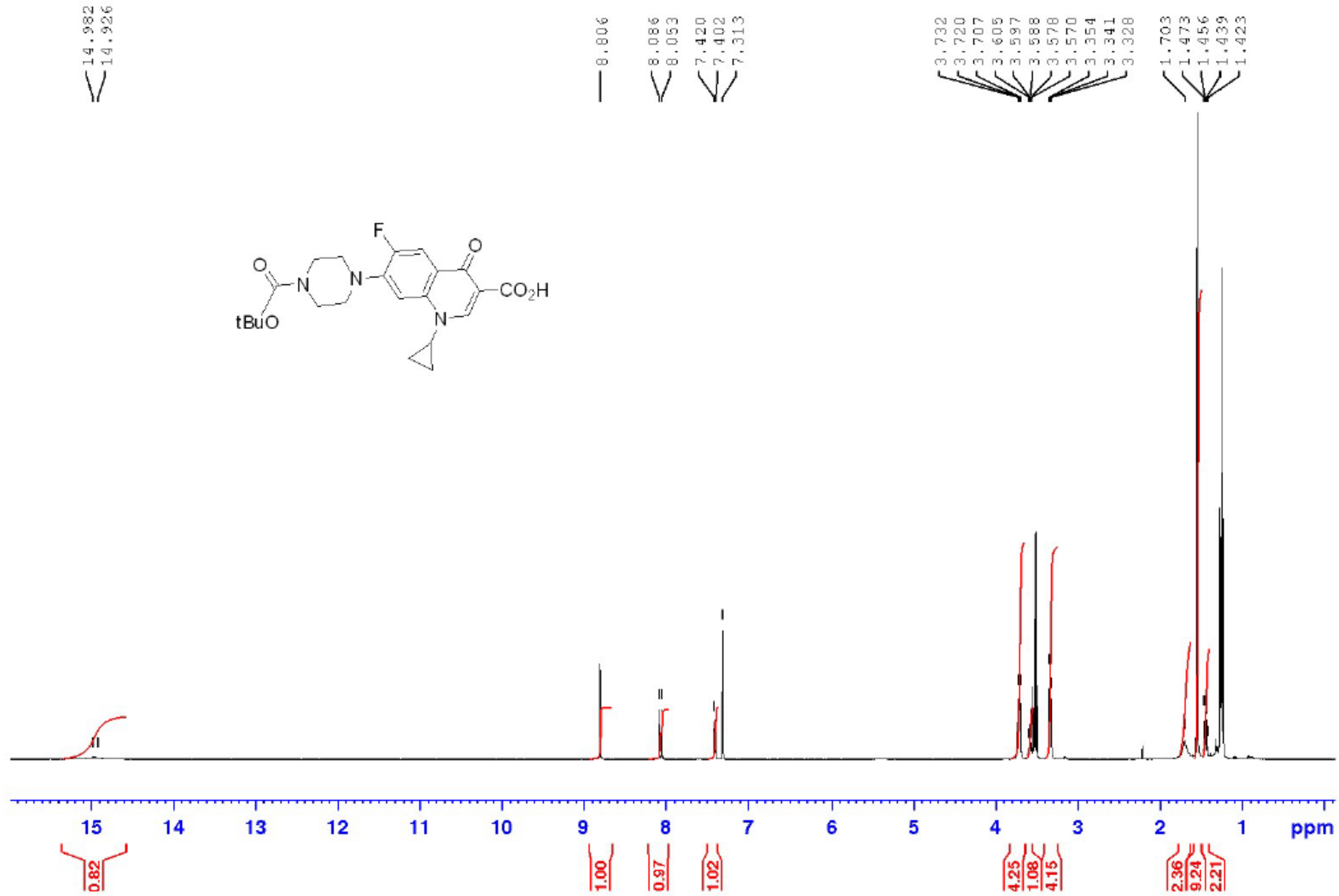

Supplement: Supplementary file 3 — Supplementary Data 1 [file 42004_2023_852_MOESM3_ESM.pdf]
